# *Francisella tularensis* induces Th1 like MAIT cells conferring protection against systemic and local infection

**DOI:** 10.1038/s41467-021-24570-2

**Published:** 2021-07-16

**Authors:** Zhe Zhao, Huimeng Wang, Mai Shi, Tianyuan Zhu, Troi Pediongco, Xin Yi Lim, Bronwyn S. Meehan, Adam G. Nelson, David P. Fairlie, Jeffrey Y. W. Mak, Sidonia B. G. Eckle, Marcela de Lima Moreira, Carolin Tumpach, Michael Bramhall, Cameron G. Williams, Hyun Jae Lee, Ashraful Haque, Maximilien Evrard, Jamie Rossjohn, James McCluskey, Alexandra J. Corbett, Zhenjun Chen

**Affiliations:** 1 Department of Microbiology and Immunology, Peter Doherty Institute for Infection and Immunity, The University of Melbourne, Melbourne, VIC Australia; 2 State Key Laboratory of Respiratory Disease, Guangzhou Institute of Respiratory Disease, The First Affiliated Hospital of Guangzhou Medical University, Guangzhou, Guangdong China; 3 School of Medicine, Tsinghua University, Beijing, China; 4 Division of Chemistry and Structural Biology, Institute for Molecular Bioscience, The University of Queensland, Brisbane, QLD Australia; 5 Australian Research Council Centre of Excellence in Advanced Molecular Imaging, The University of Queensland, Brisbane, QLD Australia; 6 Department of Infectious Diseases, Peter Doherty Institute for Infection and Immunity, The University of Melbourne, Melbourne, VIC Australia; 7 Infection and Immunity Program and the Department of Biochemistry and Molecular Biology, Biomedicine Discovery Institute, Monash University, Clayton, VIC Australia; 8 Australian Research Council Centre of Excellence in Advanced Molecular Imaging, Monash University, Clayton, VIC Australia; 9 Institute of Infection and Immunity, Cardiff University School of Medicine, Wales, UK

**Keywords:** Antimicrobial responses, Immunization, T cells, Bacterial infection

## Abstract

Mucosal-associated Invariant T (MAIT) cells are recognized for their antibacterial functions. The protective capacity of MAIT cells has been demonstrated in murine models of local infection, including in the lungs. Here we show that during systemic infection of mice with *Francisella tularensis* live vaccine strain results in evident MAIT cell expansion in the liver, lungs, kidney and spleen and peripheral blood. The responding MAIT cells manifest a polarised Th1-like MAIT-1 phenotype, including transcription factor and cytokine profile, and confer a critical role in controlling bacterial load. Post resolution of the primary infection, the expanded MAIT cells form stable memory-like MAIT-1 cell populations, suggesting a basis for vaccination. Indeed, a systemic vaccination with synthetic antigen 5-(2-oxopropylideneamino)-6-d-ribitylaminouracil in combination with CpG adjuvant similarly boosts MAIT cells, and results in enhanced protection against both systemic and local infections with different bacteria. Our study highlights the potential utility of targeting MAIT cells to combat a range of bacterial pathogens.

## Abstract (web summary)

Mucosal-Associated Invariant T (MAIT) cells are associated with established functions during bacterial infection. Here the authors show inoculation with *Francisella tularensis* results in induction of MAIT cells associated with prototypic Th1 immunity and confer protection to systemic and local infection.

## Introduction

Microbial infections in patients with compromised immunity (e.g., organ transplant recipients), in particular with multi-drug-resistant pathogens, cost several billion dollars each year^1,2^. Bacterial sepsis is associated with a very high mortality of 30–50%^3,4^ and is estimated to cause 6 million deaths among 30 million people affected worldwide every year^5^, making it a leading cause of death in hospitals. Thus, there is great interest in the development of new preventive and therapeutic approaches, including T-cell-based vaccination^6^. Although some progress has been made in this area, complete protection by T-cell vaccines has not yet been achieved and there remains a great need to understand the generation of effective T-cell memory and responses^6^.

Mucosal-associated invariant T (MAIT) cells are an abundant αβ-T-cell subset (averaging 3% of T cells in the human blood^7^), which express semi-invariant  T cell receptors (TCRs), comprising TRAV1-2-TRAJ33/12/20 in humans^8–10^ or TRAV1-TRAJ33 (Vα19-Jα33) in mice^11^, paired with a limited TCR-β chain repertoire. MAIT cells recognize conserved vitamin B2 metabolite-based antigens (Ag) from microbes, presented by the major histocompatibility complex (MHC) class I-related protein 1 (MR1) molecule. Riboflavin biosynthesis, the source of MAIT cell Ag, is an essential and highly conserved metabolic pathway present in a diverse range of bacteria and yeasts. Indeed, we and others have shown in mouse models that MAIT cells afford a protective function against several bacterial pathogens, including *Legionella longbeachae*^12^, *Francisella tularensis*^12^, *Mycobacterium bovis* BCG^13^, pathogenic *Escherichia coli* UT189^14^, *Clostridium difficile*^15^, and *Klebsiella pneumoniae*^16^. Human in vitro studies demonstrated reactivity of MAIT cells to an even broader range of species, reviewed in ref. ^17^, including *Streptococcus* spp^18,19^, *Shigella flexneri*^20^, and fungal pathogens *Aspergillus fumigatus*^21^ and *Candida albicans*^22^. Alterations in MAIT cell frequency and function have been reported in patients with tuberculosis^23–25^ and other infectious diseases^26–30^. Sandberg and colleagues^27^ reported a patient with cystic fibrosis affected by recurrent, and eventually fatal, bacterial infections, which correlated with a striking lack of detectable circulating MAIT cells.

Despite the presence of MAIT cells at many sites^31^, including high numbers in the liver^32^, female reproductive tract^33^, and intestines^34^, previous studies have mostly examined their protective role in local infections^12,13^. Thus far, a protective role has been demonstrated most convincingly in respiratory infections^12,35^, with little definitive in vivo evidence to date of their capacity to contribute to protective immunity against systemic infection. *F. tularensis* causes both respiratory disease and tularemia, a rare but often fatal systemic infection. Infection can occur via the respiratory route, by ingestion of contaminated water or via tick bites^36^. *F. tularensis* live vaccine strain (LVS) was developed as a vaccine strain with reduced pathogenicity^37^. Although its use in humans was discontinued due to the risks associated with administration of a live organism with undetermined genetics, *F. tularensis* LVS provides a useful model for pathogenicity and immune response research^38^, and it was previously shown that MAIT cells have a protective role during respiratory infection^12,39^.

Here we examined the role of MAIT cells in a systemic model of *F. tularensis* LVS infection. The kinetics and phenotype of the MAIT cell response were investigated in multiple organs. MAIT cells were highly responsive, predominantly secreting type-1 cytokines, and were not only critical to controlling bacterial load, but also formed a memory-like population after infection, which remained at high numbers, and exhibited an altered transcriptome and cell surface phenotype. Vaccination of naive mice with synthetic 5-(2-oxopropylideneamino)-6-d-ribitylaminouracil (5-OP-RU) Ag in combination with CpG adjuvant recapitulated this expanded MAIT-1 cell population and afforded better control of subsequent systemic and local infections by both *F. tularensis* and *L. longbeachae*.

## Results

### *F. tularensis* LVS activates MAIT reporter cells in vitro in an MR1-dependent manner and induces a systemic MAIT cell response in vivo

**Fig. 1:**
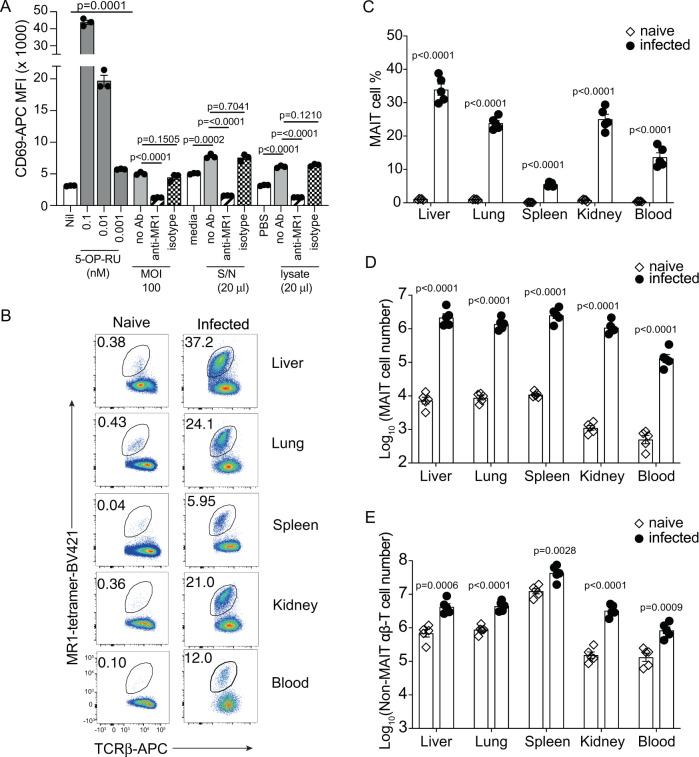
*F. tularensis* LVS activates MAIT reporter cells via MR1 in vitro and induces systematic MAIT cell expansion in vivo. **A** Jurkat.MAIT-AF7 and C1R.MR1 cells were co-incubated for 16 h with indicated amounts of 5-OP-RU, *F. tularensis* lysate (in PBS), PBS, *F. tularensis* culture supernatant, or media control (BHI). For infection samples, we co-cultured Jurkat.MAIT-AF7 for 16 h with C1R.MR1 cells that had been infected at the indicated MOI for 4 h, in the presence of gentamicin. Activation was detected by staining with anti-CD69 antibody. Anti-MR1 antibody (26.5) or isotype control (W6/32) was added, as indicated, to C1R.MR1 cells 2 h prior to co-incubation with Jurkat.MAIT cells. Experiment was performed on two separate occasions with similar results. Data show mean fluorescence intensity (MFI) (mean ± SEM of triplicate wells from one experiment). Statistical tests: unpaired *t*-test (two-tailed) comparing S/N, lysate, and MOI 100 groups with their respective controls, and one-way ANOVA with Dunnett’s multiple comparisons, comparing anti-MR1 and isotype control with unblocked samples. *P*-values are indicated. **B** Flow cytometry plots and **C** MAIT cell percentage in αβ-T cells from the liver, lung, spleen, kidney, and blood (200 μl) of C57BL/6 mice either uninfected or intravenously infected with 10^4^ CFU *F. tularensis* LVS for 14 days. **D**, **E** Absolute numbers of MAIT cells and non-MAIT αβ-T cells, respectively, in the liver, lung, spleen, kidney, and blood (200 μl) of C57BL/6 mice either uninfected or intravenously infected, 14 days post infection. The experiment was performed twice with similar results. Data show mean ± SEM *n* = 5 mice from one experiment. Statistical tests: unpaired *t*-test (two-tailed) comparing uninfected with infected mice. *p*-values are indicated. See also Supplementary Figs. 1 and 2. Source data are provided as a Source Data file.

To confirm that *F. tularensis* LVS, a riboflavin biosynthesis proficient bacteria as shown in the database Kyoto Encyclopedia of Genes and Genomes, can stimulate MAIT cells, we used an in vitro activation assay with Jurkat.MAIT.AF7 reporter cells co-cultured with Class I reduced (C1R) Ag-presenting cells expressing MR1 (C1R.MR1 cells) as antigen presenting cells, as previously described^10^. Both *F. tularensis* culture supernatant and lysate were able to stimulate Jurkat.MAIT cells in this assay as assessed by the upregulation of cell surface CD69 expression (Fig. 1A). In addition, C1R.MR1 cells infected for 4 h with a multiplicity of infection (MOI) of 100 could stimulate activation of Jurkat.MAIT cells (Fig. 1A). Anti-MR1 monoclonal antibody (mAb) (26.5), but not an isotype control mAb, completely blocked this response, consistent with MR1-dependent activation. Similar to previous studies using other bacteria^25,40^, these data demonstrated that *F. tularensis* LVS is capable of producing Ags that stimulate MAIT cells in an MR1-dependent manner.

To examine the MAIT cell response in vivo, C57BL/6 mice were infected with *F. tularensis* LVS intravenously (i.v) with a sublethal dose of 10^4^ colony-forming units (CFU). MAIT cell frequencies and numbers were assessed in a range of organs (the liver, lungs, spleen, kidneys) and in the blood by MR1-5-OP-RU tetramer staining (for gating strategy, see Supplementary Fig. 1A). Significant MAIT cell accumulation was observed in all organs tested and in the blood at 14 days post infection (dpi), compared to uninfected mice (Fig. 1B–D). We also observed an increase in numbers of MAIT cells in the small intestine (SI), from the very small numbers detectable in naive mice to ~10% and 15% of αβ-T cells in intra-epithelial lymphocyte (IEL) and lamina propria lymphocyte (LPL) preparations, respectively, at day 6 post infection (Supplementary Fig. 1B–D). Non-MAIT αβ-T cells were also increased in response to infection, as expected (Fig. 1E and Supplementary Fig. 1E), but their increase was less pronounced than that observed for MAIT cells. We next tested the MR1 dependency of MAIT cell accumulation following infection, using a blocking anti-MR1 mAb. Consistent with our previous study, which examined *Salmonella* Typhimurium infection of mice^40^, the accumulation of MAIT cells in the lungs in response to *F. tularensis* LVS was blocked with the addition of MR1-specific mAb, but not with an isotype control mAb, indicating that their accumulation was dependent on MR1-mediated activation (Supplementary Fig. 2). There was no difference in the numbers of responding non-MAIT αβ-T cells in the presence of MR1-specific mAb vs. isotype control mAb.

As *F. tularensis* infection can occur naturally through inhalation, wounds, or tick bites, we next tested various infection routes: intratracheal (i.t.), intraperitoneal (i.p.), and subcutaneous (s.c.), using a range of doses for each. Regardless of the infection route, systemic dissemination occurred resulting in a significant MAIT cell accumulation in all tested organs in mice infected with sufficient dose (Supplementary Fig. 3). Due to the ease of consistent and accurate delivery of inoculum and rapid dissemination of bacteria, we chose the i.v. route for all following infection experiments.

### The MAIT cell expansion following *F. tularensis* infection is rapid and long-lasting in all organs

**Fig. 2:**
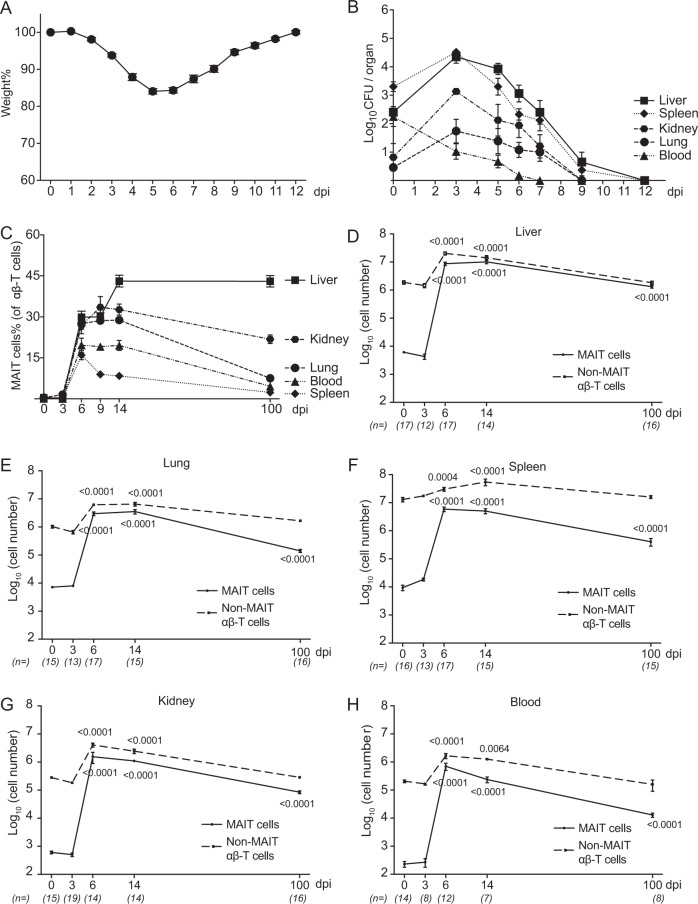
Systemic and long-lasting expansion of MAIT cells in *F. tularensis* LVS infection. **A** Body weight was recorded daily and calculated in percentage relative to starting weight of the individual mouse. Data are presented as mean ± SEM (*n* = 20 mice). **B** Bacterial load (CFU/organ or CFU/100 μl blood) from C57BL/6 mice at indicated time points post infection i.v. with 2 × 10^4^ CFU *F. tularensis* LVS. Data are presented as mean ± SEM. *n* = for day 0, 3, 5, 6, 7, 8, and 12: liver 4, 14, 8, 14, 8, 10, 6; spleen 4, 14, 8, 18, 6, 16, 6; kidney 4, 6, 8, 11, 11, 6, 6; lung 5, 14, 8, 20, 9, 16, 6; blood 4, 13, 8, 14, 8, 7, 6 individual mice. **C**–**H** Relative and absolute number of MAIT cells and non-MAIT αβ-T cells detected in various organs (as indicated) from naive and infected mice at indicated time points post infection. Pooled data from three independent experiments (mean ± SEM, *n* = 7–17 mice per group, as indicated). Statistical test: one-way ANOVA comparing each time point with 0 dpi, *p*-values are indicated. Source data are provided as a Source Data file.

Upon *F. tularensis* LVS infection, a typical valley-shaped weight change was recorded in infected mice (Fig. 2A), with mice losing weight until day 5, then recovering and reaching their starting weight by day 12. The bacteria clearance kinetics showed an initial decline in the bacterial load in the blood, but accumulation at early stages in all other sites, which reached a plateau around 3 dpi, followed by clearance (undetectable level) within 12 days (Fig. 2B).

We next investigated the kinetics of MAIT cell accumulation in various organs. Both the frequency (relative to total αβ-T cells, Fig. 2C) and number (Fig. 2D–H) of MAIT cells increased significantly in the early stages of the infection time course. Remarkably, by day 6, MAIT cells represented ∼29% of αβ-T cells in the liver, 27% in the lungs, 26% in the kidneys, 19% in the blood, and 16% in the spleen. MAIT cell numbers plateaued at about 6 dpi in all organs. Interestingly, high frequencies and numbers of MAIT cells (43% in the liver, 8% in the lungs, 21% in the kidneys, 4% in the blood, and 2% in the spleen) were maintained at 100 dpi, long after the clearance of bacteria to undetectable numbers. This was in contrast to non-MAIT αβ-T cells, which also expanded in response to infection, but then contracted as expected to their homeostatic level (in numbers) in all organs (Fig. 2D–H).

### *F. tularensis* infection polarizes MAIT cells to a MAIT-1 (Th1-like) functional phenotype

**Fig. 3:**
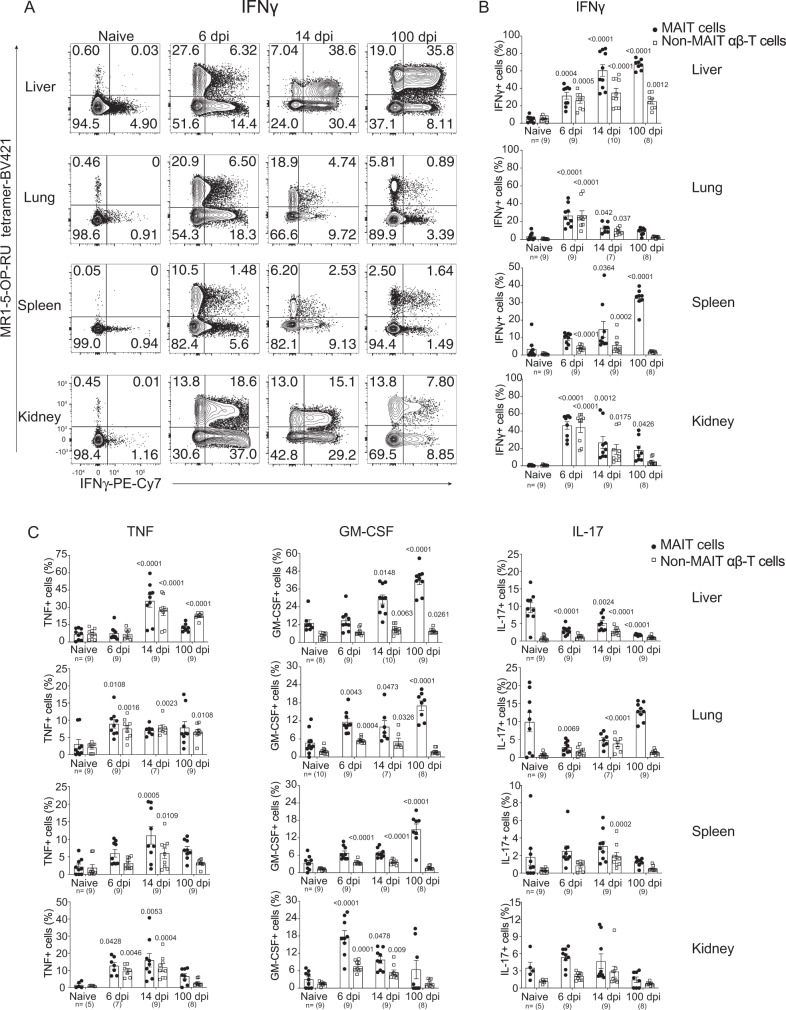
Cytokine profiling of MAIT cells upon *F. tularensis* LVS infection. **A** Representative flow cytometry plots showing intracellular cytokine staining for IFNγ of TCRβ^+^ lymphocytes from the liver, lungs, spleen, and kidneys of uninfected and infected mice on 6, 14, and 100 dpi with 2 × 10^4^ CFU *F. tularensis* LVS i.v, after 4 h of culture with brefeldin A and PMA/ionomycin stimulation. **B**, **C** Percentages of **B** IFNγ and **C** TNF, GM-CSF, and IL-17-producing cells of gated MAIT cells and non-MAIT αβ-T cells from the liver, lungs, spleen, and kidneys from mice in **A**. Pooled data of two independent experiments with similar results (mean ± SEM, *n* = 5–10 mice per group, as indicated). Statistical tests: one-way ANOVA comparing percentages of cytokine-producing MAIT cells or non-MAIT αβ-T cells at indicated time points post infection with the matching uninfected groups. *P*-values < 0.05 are indicated. Source data are provided as a Source Data file.

We next examined the functional phenotype of the MAIT cell populations that were expanded during *F. tularensis* infection by assessing their cytokine profile and expression of transcription factors (TFs) in naive mice, and at representative time points of acute infection (6 dpi), recent bacterial clearance (14 dpi), and memory phase (40 or 100 dpi). Four cytokines; tumor necrosis factor (TNF), interferon-γ (IFNγ), granulocyte-macrophage colony-stimulating factor (GM-CSF), and interleukin-17 (IL-17), previously shown to be produced by MAIT cells^35,40^, were examined directly ex vivo (without any further stimulation) (Supplementary Fig. 5), as well as following stimulation with phorbol 12-myristate 13-acetate (PMA)/ionomycin (Fig. 3 and Supplementary Fig. 4). Similar patterns were seen with both methods. MAIT cells were found to be polarized to a Th1-like phenotype, with highest proportions, and numbers, of cells producing TNF, IFNγ, and GM-CSF, but few detectable IL-17-producing cells during the acute phase of infection (6 dpi) (Fig. 3 and Supplementary Figs. 4 and 5). Interestingly, MAIT cells appeared to produce these Th1 cytokines in an organ-specific pattern, with kinetic differences in the proportions of cells producing each cytokine. This was particularly apparent late in infection, with consolidation and persistence of the population of MAIT-1 cells in the liver and spleen, but with a lower proportion of MAIT-1 cells in the lung and kidney (Fig. 3B, C). Functional polarization was most evident for IFNγ-producing MAIT cells (Fig. 3B), with TNF-producing MAIT cells displaying a similar but less pronounced pattern (Fig. 3C) and the abundance of GM-CSF-producing MAIT cells increasing throughout the infection in all organs, except for the kidney (Fig. 3C). In contrast, the proportion of IL-17-producing MAIT cells declined in both the liver and lungs (Fig. 3C), but was later increased in the lungs. The frequencies of cytokine-producing non-MAIT αβ-T cells were similar to those of MAIT cells at early time points, but lower at later times. For both MAIT cells and non-MAIT T cells, IFNγ- and TNF-producing cells were the most abundant of the cytokines tested (Fig. 3 and Supplementary Figs. 4 and 5).

**Fig. 4:**
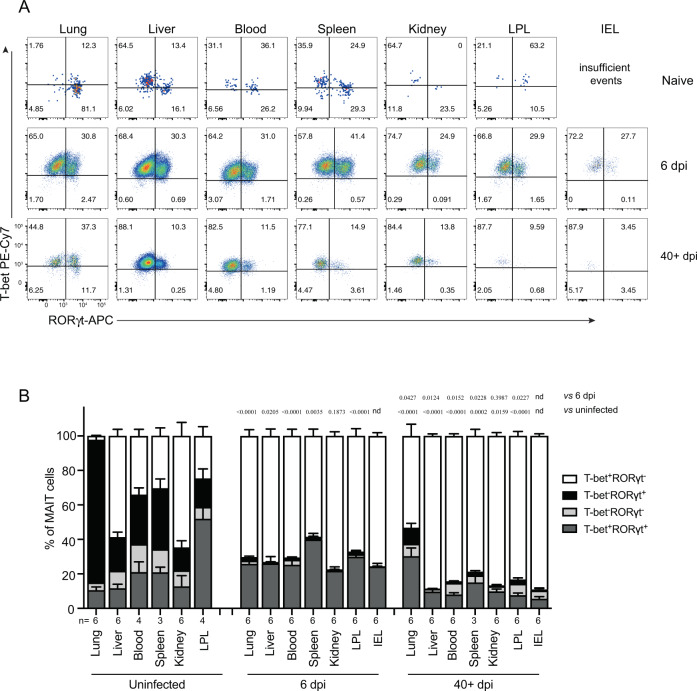
MAIT cells were polarized to functional MAIT-1 phenotype upon *F. tularensis* infection. **A** Representative flow cytometry plots showing intranuclear staining for T-bet (representing Th1) and RORγt (Th17) in gated MAIT cells from the liver, lungs, spleen, kidneys, LPL, IEL, and blood of naive and infected mice on 6 and 40+ dpi (41 and 68 dpi in two experiments) with 10^4^ CFU *F. tularensis* LVS i.v. Numbers in quadrants represent cell percentage. It is noteworthy that IEL from naive mice yielded insufficient numbers of MAIT cells for accurate assessment of transcription factor expression and, thus, were omitted from our analysis. **B** Percentage of MAIT cells expressing combinations of T-bet and RORγt from the same mice in **A**. Pooled data from two independent experiments (mean ± SEM, *n* = 3–6 mice per group, as indicated). One-way ANOVA with Tukey’s multiple comparisons test was performed on MAIT-1% in each organ (except for IEL) between time points as indicated; *p*-values are indicated, nd; not determined. Source data are provided as a Source Data file.

The production of Th1 and Th17 cytokines by MAIT cells was consistent with their expression of the closely correlated TFs T-bet and RORγt, respectively. We found that naive pulmonary MAIT cells were predominantly (>80%) T-bet^−^ RORγt^+^ (MAIT-17) (Fig. 4), consistent with our previous study^35^, whereas in the liver ~60% had a MAIT-1 phenotype (T-bet^+^RORγt^−^) and there was a mixture of MAIT-1 and MAIT-17 cells in the kidney, spleen, and blood (Fig. 4). Following infection (6 dpi) with *F. tularensis*, a greater proportion of MAIT cells was T-bet^+^RORγt^−^ in all organs and in the blood (Fig. 4). Remarkably, after 40 dpi, long after bacteria were cleared to undetectable levels (Fig. 2B), the phenotype of MAIT cells remained skewed towards MAIT-1 (Fig. 4).

We next examined the expression of several markers associated with memory T cells, by MAIT-1 cells from the liver of naive mice and mice with recently cleared infection (14 dpi) or long-term post infection (60+ dpi) (Supplementary Fig. 6). In contrast to conventional CD8^+^ T cells, MAIT cells are CD44^hi^, CD62L^lo^ and express CD69 in naive mice, and the expression of these markers did not change following infection (Supplementary Fig. 6). MAIT cells upregulated expression of IL-18R and CD49a by day 14 post infection and these remained high after 60 days. CD127 (IL-7R) was also increased on MAIT cells at 60 dpi, consistent with its expression on long-lived memory T cells^41^, whereas CD69 was expressed bimodally (Supplementary Fig. 6). These data are consistent with the development of a long-lived memory-like MAIT cell pool after infection.

### MAIT cells exhibit substantially altered transcriptomes following infection with *F. tularensis*

**Fig. 5:**
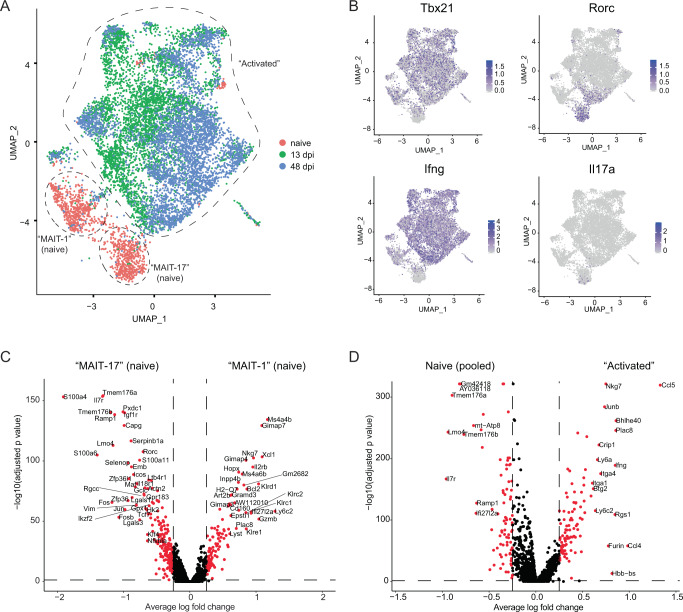
Droplet-based scRNA-seq analysis of liver MAIT cells before and upon *F. tularensis* LVS infection. **A** UMAP representation of post-QC liver MAIT cell transcriptomes from naive mice (*n* = 17) and mice at days 13 (*n* = 2) and 48 (*n* = 4) post infection, calculated using principal components (PC) 1–30. **B** UMAP representation of *Tbx21*, *Rorc*, *Ifng*, and *Il17a* expression. **C**, **D** Volcano plots of differential gene expression calculated based on unsupervised clustering (Supplementary Fig. 7B), average log fold changes below −0.25 or above 0.25 are highlighted in red, and gene names were highlighted when the average log fold change was below −0.6 or above 0.6. **C** Volcano plots of differential gene expression analysis between MAIT-1 (cluster 5) and MAIT-17 (cluster 7) cells from naive mice. **D** Volcano plots of differential gene expression analysis between MAIT cells prior to (clusters 5 and 7) and post infection (clusters 0, 1, 2, 3, 4, 6, 8, 9, 10, 11, and 13). Cluster numbering derived from Supplementary Fig. 7B.

To investigate at genome-scale whether cell-intrinsic changes had occurred to MAIT cells during infection, we performed droplet-based single-cell RNA sequencing (scRNA-seq) on cells sorted from the liver prior to and at days 13 and 48 post infection. After quality control (based on numbers of unique molecular identifiers, numbers of genes, and % mitochondrial reads), we retained 1276, 4512, and 4289 cell numbers for naive, day 13, and day 48 samples, respectively (Supplementary Fig. 7A–C). Unsupervised clustering resulted in 17 different clusters, two of which showed low numbers of expressed genes and high mitochondrial content, whereas another cluster had high expression of Ag-presenting cell-related genes (including *CD74*, *H2-Eb1*, *H2-Aa*, *H2-Ab1*). Therefore, these three clusters were excluded from subsequent analysis (Supplementary Fig. 7B). Following principal component analysis (PCA), Uniform Manifold Approximation and Projection (UMAP), using two (Fig. 5A) or three dimensions (https://haquelab.mdhs.unimelb.edu.au/MAIT_Francisella/), revealed two distinct MAIT cell populations present in naive samples. These tended to express either *Tbx21* and *Ifng* (encoding T-bet and IFNγ), supporting classification as MAIT-1 cells, or *Rorc* and *Il17a* (encoding RORγt and IL-17A), supporting classification as MAIT-17 cells (Fig. 5B). Crucially, MAIT cells at 13 dpi exhibited substantially altered transcriptomes compared to naive MAIT cells, consistent with these cells having acquired an activated state during infection (Fig. 5A). Moreover, transcriptomes of MAIT cells remained substantially different at 48 dpi from those of naive MAIT cells, confirming that molecular changes had been preserved in MAIT cells during infection (Fig. 5A). Consistent with flow cytometry analysis of T-bet/RORγt (Fig. 4), the vast majority of MAIT cells detected during infection exhibited *Tbx21* but not *Rorc* expression, consistent with a skewing towards a MAIT-1 response, and not MAIT-17 (Fig. 5B).

Unsupervised clustering highlighted not only the presence of two naive MAIT cell clusters but a large number of activated MAIT cell clusters from day 13, suggesting a substantial increase in heterogeneity in MAIT cells induced by *F. tularensis* LVS infection, which was largely preserved by day 48 (Supplementary Fig. 7B). To determine molecular differences between the two naive MAIT cell clusters, differential gene expression analysis (DGEA) was conducted between these two clusters alone. This revealed a number of significantly upregulated genes associated with either phenotype, including *Nkg7* and *Gzmb* in MAIT-1 cells, and *Ilr7*, *Icos*, and *Rorc* in MAIT-17 cells (Fig. 5C and Supplementary Table 1). To broadly define molecular changes induced by infection, DGEA was conducted between pooled naive (Cluster 5 + 7) vs. activated (all remaining clusters, except 12 and 14) MAIT cell clusters. We noted that many genes were upregulated and preserved after infection, including *Nkg7*, *Ccl5*, *Ifng*, and *Ccl4* (Fig. 5D and Supplementary Table 2), which reinforces that MAIT cells had acquired an altered cellular state. Thus, liver MAIT cells exist in two distinct cellular states in naive mice, which, upon *F. tularensis* LVS infection, undergo substantial heterogeneous cellular change, preserved for several weeks.

### MAIT cells are critical for controlling bacteria during *F. tularensis* systemic infection

**Fig. 6:**
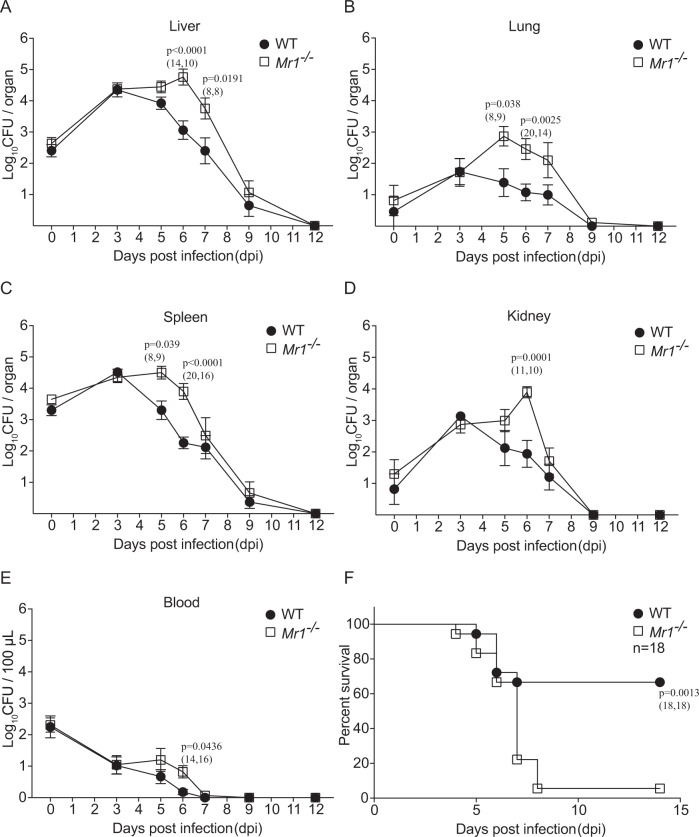
Systemic control of bacterial load and survival after intravenous *F. tularensis* infection are enhanced in the presence of MAIT cells. **A**–**E** WT (C57BL/6) or *Mr1*^−/−^ mice were infected i.v. with 2 × 10^4^ CFU *F. tularensis* LVS. The bacterial burden (CFU) in the liver, lung, spleen, kidney, and blood (100 μl) was assessed at the indicated dpi. 0 dpi was examined at 4 h after inoculation. Pooled data (mean ± SEM) from two independent experiments with similar results. Two-way ANOVA on log-transformed data. *P*-values < 0.05 are indicated. Numbers in brackets indicate number of mice: *n* = (WT, *Mr1*^−/−^). **F** Survival of WT (C57BL/6) or *Mr1*^−/−^ mice (*n* = 18 mice per group) challenged with 2.5 × 10^4^ CFU *F. tularensis* LVS. Log-rank test. *P*-value is indicated. Source data are provided as a Source Data file.

Next, we examined the protective role of MAIT cells in *F. tularensis* systemic infection by comparing wild-type (WT) (C57BL/6) mice with *Mr1*^−/−^ mice, which lack MAIT cells. Following inoculation of mice i.v. with 10^4^ CFU *F. tularensis* LVS, significantly higher bacterial loads were observed at 6 dpi in all organs (the liver, lung, spleen, and kidney) and blood of *Mr1*^−/−^ mice than in WT mice, and the difference was also significant at 5 and 7 dpi in some organs (Fig. 6A–E). In these experiments, bacteria were cleared to undetectable levels by day 12 post infection either with or without MAIT cells. However, when a higher infection dose (2.5 × 10^4^ CFU) was used, *Mr1*^−/−^ mice showed significantly poorer survival (based on humane endpoints outlined in methods) (Fig. 6F), suggesting that MAIT cells play a protective role, which can be critical for controlling bacterial growth when other arms of the immune system are intact. Consistent with this protective role of MAIT cells, *Mr1*^−/−^ mice that succumbed to infection (reached humane endpoints, thus necessitating euthanasia) had significantly higher bacterial loads in the liver and spleen than matched WT mice culled at the same time points (Supplementary Fig. 8).

We next tested the protective role of MAIT cells in a more severe infection setting. The peritoneal cavity contains only a small number of immune cells^42^ and i.p. inoculation of WT mice with *F. tularensis* can cause severe infection and death in some mice with as few as 10 CFU (Supplementary Fig. 9B). Here, WT mice received adoptive transfer of MAIT cells, followed by bacterial challenge with 10 CFU *F. tularensis* LVS (Supplementary Fig. 9A, B). Transferred MAIT cells were sorted from the livers of mice infected with *F. tularensis* to boost MAIT cell numbers. When adoptively transferred, these cells provided a dose-dependent protection with full protection achieved when 5 × 10^5^ MAIT cells were transferred (Supplementary Fig. 9B).

**Fig. 7:**
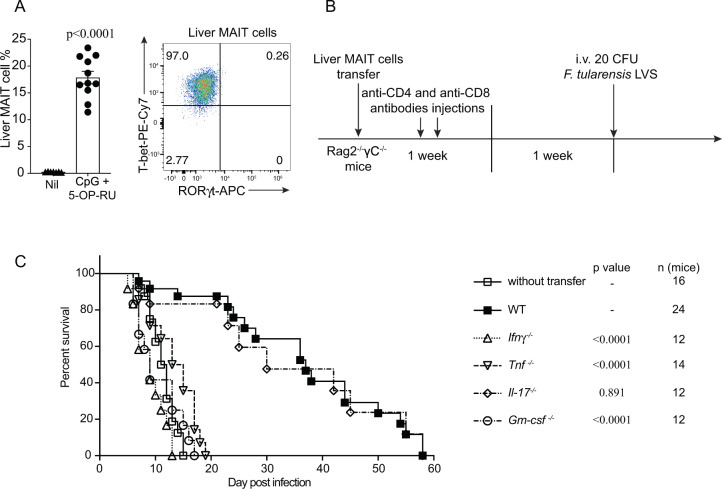
MAIT cell-mediated protection in immune-compromised mice requires IFNγ, TNF, and GM-CSF. **A** MAIT cell percentage of αβ-T cells in the liver and representative FACS plot showing intranuclear staining for T-bet and RORγt of MAIT cells from donor C57BL/6 mice vaccinated with CpG and 5-OP-RU i.v. for 7 days, prior cell sorting for adoptive cell transfer. Pooled data from 7 (nil) or 11 (vaccinated) mice from 3 independent experiments (mean ± SEM). Unpaired *t*-test (two-tailed). *P* < 0.0001. **B** Schematic of protocol for MAIT cell adoptive transfer and *F. tularensis* LVS challenge: 10^5^ liver MAIT cells from C57BL/6 (WT, shown in **A**), *Ifnγ*^−/−^, *Tnf*^−/−^, *Gm-csf*^−/−^, or *Il-17*^−/−^ mice vaccinated with CpG (10 nmol) and 5-OP-RU (2 nmol) i.v. for 7 days were sorted by flow cytometry and transferred i.v. into *Rag2*^−/−^*γC*^−/−^ mice. The mice were treated with anti-CD4 and anti-CD8 mAb injection (i.p., 0.1 mg each) at days 1 and 3 post MAIT cell transfer, to deplete contaminating conventional T cells. After 2 weeks, mice were infected with an otherwise lethal dose (20 CFU) of *F. tularensis* LVS i.v. **C** Survival of untreated *Rag2*^−/−^*γC*^−/−^ mice or *Rag2*^−/−^*γC*^−/−^ mice following transfer of MAIT cells from WT, *Ifnγ*^−/−^, *Tnf*^−/−^, *Gm-csf*^−/−^, or *Il-17*^−/−^ mice according to schematic shown in **B**. Pooled data from two independent experiments with similar results (*n* = 12–24 mice per group, as indicated). Log-rank tests (*Ifnγ*^−/−^, *Tnf*^−/−^, *Gm-csf*^−/−^, and *Il-17*^−/−^ groups were compared with WT group). *P*-values are indicated. Source data are provided as a Source Data file.

To further test the capacity for MAIT cells to provide protection, we then turned to an immune-compromised setting and utilized *Rag2*^−/−^*γC*^−/−^ mice, which lack T, B, and natural killer (NK) cells^43^. These mice are severely immune compromised and, as expected, all mice succumbed to a low dose of *F. tularensis* LVS (20 CFU) i.v. within 20 dpi (Fig. 7C). For these experiments, we sourced MAIT cells for adoptive transfer from the livers of WT mice primed with synthetic 5-OP-RU Ag and CpG adjuvant (Fig. 7A), to ensure no carry over of live bacteria to the recipient mice before challenge. This priming method recapitulated the predominant MAIT-1 phenotype in the liver as seen with *F. tularensis* infection (Fig. 7A). Mice receiving adoptively transferred MAIT cells were able to significantly prolong survival after subsequent i.v. challenge with *F. tularensis* LVS, compared to mice without MAIT cell transfer (Fig. 7A–C), suggesting that MAIT cells can indeed provide protection in their own right, in the absence of several other immune elements. To further explore the role of individual cytokines produced by MAIT cells in protecting against systemic *F. tularensis* LVS infection, adoptive transfer of WT vs. cytokine-deficient MAIT cells to *Rag2*^−/−^*γC*^−/−^ mice was performed. WT or IL-17-deficient MAIT cells were able to significantly prolong survival of mice following infection, with some mice surviving up to 58 dpi (Fig. 7C). In contrast, MAIT cells deficient in the production of TNF, IFNγ, or GM-CSF were unable to provide protection, with mice succumbing to infection similar to the untreated *Rag2*^−/−^*γC*^−/−^ mice. This was consistent with the susceptibility of mice genetically deficient in individual cytokines (Supplementary Table 3). Together, these data show that MAIT cells have the capacity to contribute to systemic protection against bacterial infection and this can be the difference between life and death in the absence of other arms of the immune system.

### MR1 deficiency has minimal impact on innate cells during *F. tularensis* infection

As previous studies have suggested a role for MAIT cells in the recruitment of other cell types in infections with *F. tularensis* and other bacterial pathogens^12,39,44^, we next examined the response of a panel of innate immune cells by comparing *Mr1*^−/−^ and WT mice at early time points post infection. Specifically, we assessed neutrophils (Ly6G^+^CD11b^+^), NK cells (NK1.1^+^CD49b^+^TCRβ^−^), macrophages (CD11c^+^F4/80^+^), CD11b^+^, and CD103^+^ dendritic cells (MHCII^high^CD11c^high^), in both the liver and lungs at day 3 and 5 post infection. Consistent with the findings of others^12,39^, we observed small, but statistically significant, differences in the numbers of some innate cells in the presence and absence of MR1 (Supplementary Fig. 10). Specifically, there were more NK cells (3 dpi), neutrophils (5 dpi), and macrophages (5 dpi) in the lungs of WT mice than in *Mr1*^−/−^ mice, suggesting a role of MAIT cells in recruiting innate cells. However, no difference was observed in innate cells in the livers between WT and *Mr1*^−/−^ mice at either time point. It should be noted that *Mr1*^−/−^ and WT mice have similar bacterial loads at day 3, whereas by day 5 the bacterial load is higher in *Mr1*^−/−^ mice (Fig. 6), suggesting the recruitment of macrophages and neutrophils was not simply due to an increase in bacteria.

### Systemically boosted MAIT cells manifest vaccination potential against local and systemic pathogens

**Fig. 8:**
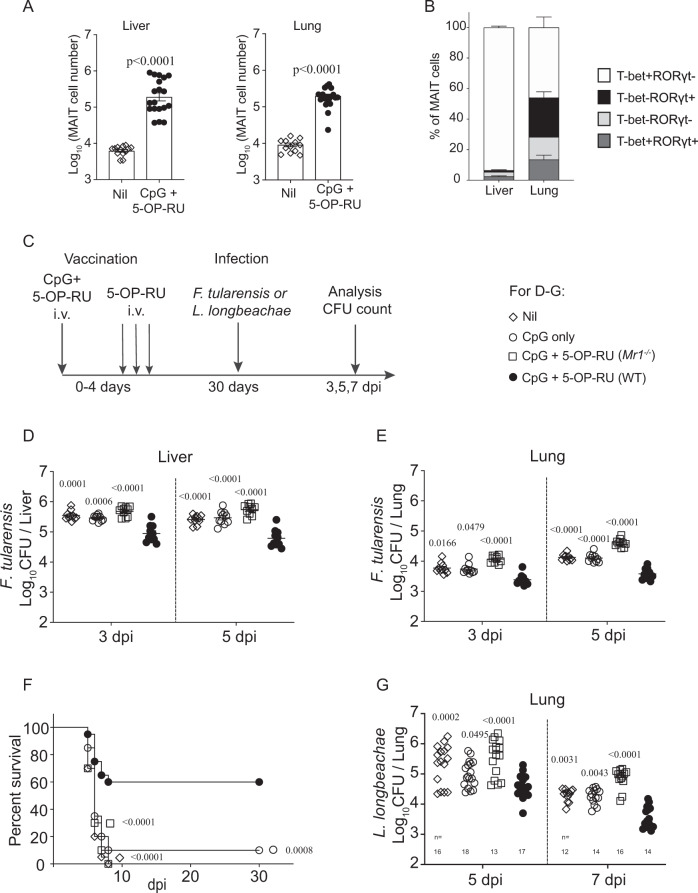
MAIT cell-targeted vaccination delivers significant protection against both local and systemic bacterial pathogens. **A** MAIT cell number, and **B** T-bet and RORγt phenotype of MAIT cells in the liver and lungs of C57BL/6 mice 30 days after i.v. vaccination with 5-OP-RU and CpG (as depicted in **C**). Pooled data from three independent experiments with similar results (mean ± SEM. **A**: *n* = 13 (unvaccinated) or 19 (CpG + 5-OP-RU) mice per group, **B**: *n* = 9 mice per group). Unpaired *t*-test (two-tailed), *P*-values are indicated. **C** Schematic flow chart for vaccination and challenge. Mice were administered with CpG (10 nmol) alone or CpG together with 2 nmol 5-OP-RU in 200 μl PBS at day 0) and 5-OP-RU (2 nmol in 200 μl PBS on day 1, 2, and 4). After 30 days, mice were infected with 2.5 × 10^4^ CFU of *F. tularensis* LVS i.v. or 10^4^ CFU of *L. longbeachae* i.n. **D**, **E** Bacterial load in the liver and lungs, respectively, of unvaccinated WT (C57BL/6) mice (Nil, open diamond), mock-vaccinated WT mice (CpG-only, open circle), vaccinated *Mr1*^−/−^ mice (CpG + 5-OP-RU, open square), and vaccinated WT mice (CpG + 5-OP-RU, filled circle) at indicated time points post *F. tularensis* challenge (2.5 × 10^4^ CFU, i.v.). **F** Survival of mice vaccinated (as in **D**–**F**) after *F. tularensis* challenge (3 × 10^4^ CFU, i.v.). **G** Bacterial load in lungs of mice vaccinated and challenged with *L. longbeachae* at indicated time points post *L. longbeachae* challenge. One-way ANOVA on log-transformed data (unvaccinated, CpG-only, and vaccinated *Mr1*^−/−^ groups were compared with vaccinated WT group). *P*-values are indicated. Pooled data from three independent experiments with similar results (mean ± SEM, *n* = 10 (**D**, **E**), 20 (**F**), or 12–18, as indicated (**G**) mice per group). Source data are provided as a Source Data file.

We previously showed that MAIT cells can be primed locally in the lungs, providing more rapid control of *L. longbeachae* infection in the lungs^35,45^. In the *Legionella* infection model, the majority of MAIT cells were MAIT-17^45^. Here, an immunization scheme was developed to systemically boost MAIT-1 cells as a component of vaccination. Modifying our previously reported methods^35,44,45^, we used synthetic MAIT Ag 5-OP-RU administered i.v. together with CpG adjuvant (Fig. 8A). This resulted in a significantly expanded MAIT cell pool systemically (in liver, lungs, and spleen) without significantly changing the total numbers of non-MAIT αβ T cells (Fig. 8A and Supplementary Fig. 11A, B). Furthermore, the majority of MAIT cells boosted with this vaccination scheme had a MAIT-1 phenotype (Fig. 8B), similar to the MAIT cell phenotype driven by systemic *F. tularensis* LVS infection (Figs. 3 and 4).

To test the protective capacity afforded by MAIT cell vaccination, mice were then challenged with *F. tularensis* LVS i.v. as depicted in Fig. 8C. Vaccinated mice were rested for a month to foster MAIT memory formation and to allow the immune system to return to homeostasis. Both the bacterial load and mouse survival were assessed independently. Vaccinated WT C57BL/6 mice showed a significant reduction in the bacterial load in the liver and lungs at 3 and 5 dpi compared to unvaccinated mice, CpG-only-treated mice, or vaccinated *Mr1*^−/−^ mice (Fig. 8D, E and Supplementary Fig. 11C). Thus, our data show that a systemic vaccination scheme that targets MAIT cells can result in greater control of bacterial load at early time points after infection. Consistent with the stronger MAIT cell response, vaccinated mice also showed enhanced survival of otherwise lethal *F. tularensis* infection (Fig. 8F). We next assessed whether systemic MAIT cell boosting could provide enhanced protection when challenged with a different bacterial pathogen. For this, we used intranasal infection with *L. longbeachae*. The bacterial load in the lungs of mice at 5 and 7 days post challenge (intranasally (i.n.)) were significantly lower in vaccinated C57BL/6 mice than in the three control groups (Fig. 8G), indicating that vaccination to boost MAIT cells can provide a level of protection against unrelated bacterial pathogens.

## Discussion

Our data show that MAIT cells respond in several organs and in the blood during systemic infection of mice with *F. tularensis* LVS. Regardless of delivery route, the infection resulted in bacterial replication in several organs and a significant accumulation of MAIT cells was observed in the liver, lungs, kidney, spleen, and blood.

In an earlier study, i.n. delivery of *F. tularensis* LVS induced significant MAIT cell expansion in the lungs at day 8 post infection^12^. Although that study defined MAIT cells as CD4^−^CD8^−^ T cells, here we have precisely identified MAIT cells using MR1-5-OP-RU tetramers, allowing us to detect MAIT cell accumulation as early as 3 dpi, with the peak of MAIT cell numbers observed at day 6 post infection in most organs. MAIT cells are considered to bridge innate and adaptive immunity^46^ consistent with our finding that compared with WT mice, control of bacterial burden was delayed in *Mr1*^−/−^ mice lacking MAIT cells. However, in both WT and *Mr1*^−/−^ mice, bacteria were cleared to undetectable levels by 12 days post infection, suggesting that other immune factors, such as conventional T cells, also play an important role. Indeed, the majority of T cells producing cytokines after infection were non-MAIT T cells, presumably Ag-specific conventional T cells, or other innate T-cell subsets, which are known to respond to *F. tularensis* infection^47,48^. Importantly, in the case of severe systemic infection (i.e., with a higher inoculum dose), or in the absence of other immunity (i.e., in *Rag2*^−/−^*γC*^−/−^ mice), MAIT cell-mediated protection became of life or death importance.

Mouse MAIT cells were previously reported to egress the thymus with mutually exclusive MAIT-1 or MAIT-17 effector phenotypes^49,50^, which correlate with T-bet and RORγt expression, respectively. In naive mice, we observed a skewed composition of transcription factor expression according to tissue sites, with greater proportions of MAIT-1 cells in the liver, MAIT-17 cells in the lungs, and similar proportions of MAIT-1 and MAIT-17 cells in the kidney, spleen, and blood. Consistent with previous reports^49–51^, this observation strongly suggests tissue-specific imprints can favor one phenotype of MAIT cells. Surprisingly, infection with *F. tularensis* LVS resulted in a polarization towards a MAIT-1 response, regardless of the tissue location. For example, naive lung MAIT cells display a predominant MAIT-17 phenotype and express high levels of IL-17^35,40^. However, upon *F. tularensis* LVS infection, >60% of MAIT cells in the lungs were T-bet^+^RORγt^-^, distinct from the response to *Legionella* or *Salmonella* infection seen previously where MAIT cells from infected lungs were mostly MAIT-1/17 (T-bet^+^/RORγt^+^ double positive)^35,40^. The moderate skewing of liver MAIT cell population to MAIT-1 in naive mice was further consolidated to >85% T-bet^+^ after *F. tularensis* infection. In a previous study, elevated IL-17A gene transcription was detected in total lung tissue of WT compared to *Mr1*^−/−^ mice following pulmonary *F. tularensis* LVS infection^12^. However, here, using intracellular cytokine staining, we did not observe an increase in IL-17 production in the lungs by MAIT cells. Thus, in addition to tissue-specific cues, polarization signals from pathogens drive different MAIT cell responses. MAIT cells have been described as long lived and the increased numbers relative to naive mice, expression of markers such as CD127, and persisting altered gene expression suggest the development and maintenance of a memory pool. However, we cannot rule out that the MAIT cell population long-term post infection may also contain a small number of recent thymic emigrants. It is unclear from the current study whether polarized MAIT cell populations resulted from a preferential expansion, recruitment, or MAIT cell plasticity.

Interestingly, despite differences in the functional phenotype of MAIT cells induced by infection with *L. longbeachae*^35^ and *F. tularensis*, we saw protection against both bacteria by MAIT-1 cells following vaccination of mice. We previously showed that IFN-γ production from MAIT cells was necessary for their protective capacity in a model of *Legionella* infection in the absence of other T cells, B cells, or NK cells^35^. Here, significant proportions of MAIT cells produced IFNγ, TNF, and GM-CSF in response to *F. tularensis* infection and adoptive transfer experiments showed that all three cytokines were equally important for the ability of MAIT cells to provide protection in an immunocompromised setting. Moreover, we found that mice lacking IFNγ or TNF were more susceptible than GM-CSF-deficient mice. However, in immune-sufficient mice, the mechanisms behind the protective contribution of MAIT cells are not clear. Indeed, they do not represent the majority of T cells producing these cytokines. Previous studies suggested an indirect protection role of MAIT cell in pulmonary *F. tularensis* LVS infection, whereby MAIT cell-derived GM-CSF attracted monocyte maturation and subsequent conventional CD4^+^ T-cell recruitment^12,39^. Here we observed that GM-CSF production by MAIT cells in the lungs, spleen, and kidneys was detectable from day 6 and increased around day 14 post infection. Consistent with these previous studies, we also observed small but significant increases in innate cells, including NK cells, neutrophils, and macrophages in WT compared to *Mr1*^−/−^ mice. Thus, MAIT cells may limit bacterial load, at least in the lungs, in part by recruiting other cell types. Together, our data support a direct protection role for MAIT cells in this model where no other T cells are present, whereas in the presence of a complete immune response, the exact mechanism of MAIT cells’ protective effect is less clear, warranting further exploration.

MAIT cells have been termed effector-memory cells due to their expression, upon thymic egress, of a memory-like phenotype (CD62L^low^, CD44^high^)^49^. We show here that, upon infection, their initial response (accumulation and cytokine secretion) elicited a critical bridging protection role between innate and adaptive immunity. After the resolution of infection, an expanded MAIT cell pool was formed and maintained at significantly higher frequency among αβ T cells. This MAIT cell pool had an altered phenotype (MAIT-1), increased expression of markers associated with T-cell memory, and altered heterogeneous transcriptomes. A single vaccination reconstituted a similarly expanded MAIT cell pool post infection (i.e., boosted number of MAIT-1 cells). The vaccinated mice controlled bacterial burdens more efficiently than naive, non-boosted mice, reducing the bacterial load at day 3 and day 5 post infection. It should be noted that although we did not test the kinetics of bacterial clearance (below detection limit), vaccination reduced the number of mice that succumbed to infection but did not alter the kinetics of survival. Thus, this study shows that MAIT-1 cells have  both an innate-like rapid response and adaptive memory-like features, consistent with our previous observations on MAIT-17/1 cells from respiratory infection models with *S*. Typhimurium and *L. longbeachae*^35,40^, which amplifies our knowledge of both phenotypes of MAIT cells. Consistent with previous studies^33,51,52^, our data suggest that tissue-specific signals foster different polarization of MAIT cells at different sites. During infection, both pathogen and host tissue signals appear to drive the polarization of MAIT cells, and in the long term after infection, the resultant MAIT cells are likely to be shaped by a balance of these factors.

The augmented number and longevity of the expanded MAIT cell population and altered functional phenotype (MAIT-1) post infection support the concept of MAIT cell memory induction, and hence their potential as a vaccine target. Our vaccination and challenge data indeed provide proof-of-concept that MAIT cells that are systemically boosted in number and polarized in function can confer better protection against different pathogens at different sites: *F. tularensis* LVS systemic infection and *L. longbeachae* pulmonary infection. Our findings support MAIT cells as a target for immune interventions, including against antibiotic-resistant pathogens.

## Methods

### Mice

All mice were bred and housed in the Biological Research Facility of the Peter Doherty Institute for Infection and Immunity (Melbourne, Victoria, Australia), with a 12 h day/night light cycle (light from 7 a.m. to 7 p.m. and dark from 7 p.m. to 7 a.m.), humidity of 40–70%, and temperature of 19–22 °C. *Mr1*^−/−^ mice were generated as previously described^53^ by breeding *Vα19iCα*^−/−^*Mr1*^−/−^ mice^54^ (from Susan Gilfillan, Washington University, St. Louis School of Medicine, St Louis, MO) with C57BL/6 mice and inter-crossing of F1 mice. The genotype was determined by tail DNA PCR at the MR1 locus as previously described^53^. Mice aged 6–12 weeks were used in experiments, after approval by the University of Melbourne Animal Ethics Committee.

### Compounds, immunogens, and tetramers

5-OP-RU was prepared as described previously^55^. Ac-6-FP was purchased from Shircks Laboratories. CpG combo (CpG B and *CpG P* together), sequence: 5′-T*C*G*T*C*G*T*T*T*T*G*T*C*G*T*T*T*T*G*T*C*G*T*TT*CG*T*CG*A*CG*A*T*CG*G*C*G*CG*C*G*C*C*G-3′ (*phosphorothioate linkage) non-methylated cytosine–guanosine oligonucleotides was purchased from Integrated DNA Technologies, Singapore. Murine MR1 and β2-microglobulin genes were expressed in *E. coli* purified from inclusion bodies, and were refolded and purified as described previously^17,56^. MR1-5-OP-RU and MR1-6-FP tetramers were generated as described previously^57^.

### Bacterial strains

Cultures of *F. tularensis* LVS were grown in 10 ml of brain heart infusion (BHI) broth for 16–18 h at 37 °C with shaking at 180 r.p.m., or on Cysteine Heart Agar (CHA) plates containing 10 μg/ml ampicillin, 7.5 μg/ml colistin, and 4 μg/ml trimethoprim for 4 days. For the infecting inoculum, with the estimation that 1 OD_600_ = 2.4 × 10^9^/ml, bacteria from overnight cultures were washed and diluted in phosphate-buffered saline (PBS) for instillation to mice. A sample of inoculum was plated onto CHA plates with appropriate antibiotics (as above mentioned) for verification of bacterial concentration by counting CFU. *L. longbeachae* NSW150 was grown in buffered yeast extract (BYE) broth or agar plates supplemented with streptomycin (50 μg/ml).

Bacterial culture supernatant was taken from similarly prepared cultures after centrifugation (benchtop centrifuge: 16,200 × *g*, 1 min) and filtration through 0.2 μm filters to remove bacterial cells. To prepare lysates, the cell pellets after centrifugation, from the same cultures, were washed twice with 1 ml cold PBS and resuspended in 1 ml cold PBS. Bacteria were then lysed by repeated ultrasonication (Misonix S4000 sonicator, 50 amplitude 10 × 20 s) and the samples stored at −80 °C until use.

### In vitro activation assays

Activation of MAIT cells was assessed using a MAIT reporter assay^10,17^. C1R.MR1 cells^10^ were uninfected or infected with *F. tularensis* (MOI 10 or 100) for 4 h. These were then washed and co-cultured overnight at 10^5^ cells per well in 96-well U-bottom plates with an equal number of Jurkat.MAIT.AF7 cells^17^ (derived from the parental Jurkat cell line J.RT3-T3.5 (ATCC TIB-153)), which express a TRAV1-2/TRBV6-1 TCR^11^, in RPMI-1640 media (Gibco) containing 10% fetal calf serum (FCS) and 100 μg/ml gentamicin at 37 °C, 5% CO_2_. In separate wells, uninfected C1R.MR1 cells were co-incubated with Jurkat.MAIT cells in the presence of bacterial lysates or filtered bacterial culture supernatants from overnight cultures in BHI broth. To block MR1 presentation, anti-MR1 mAb 26.5 or isotype control mAb W6/32 was added, as indicated, to the C1R.MR1 cells at 20 μg/ml prior to co-culture with Jurkat.MAIT cells. Following overnight incubation, cells were stained with antibodies to CD3-phycoerythrin (PE)-Cy7 (UCHT1, eBioscience; 1:300), CD69-allophycocyanin (APC) (FN50, BD Biosciences; 1:25), and 7-aminoactinomycin D (7AAD; 1:500) before analysis on a BD Fortessa (BD Biosciences) flow cytometer using Diva Software (version 7, BD Biosciences). Activation of Jurkat.MAIT cells was measured by an increase in surface CD69 expression. Infection of C1R cells was confirmed by spreading an aliquot of cells on CHA plates with antibiotics (as above) and incubation for 4 days at 37 °C under aerobic conditions before counting the resultant colonies.

### Inoculation of and assessment of infected mice

*F. tularensis* LVS was delivered by i.v. injection via the tail vein (10–3 × 10^4^ CFU in 200 μl PBS), i.n. (50–200 CFU in 50 μl PBS), i.t. (10–50 CFU in 50 μl PBS), i.p. (20–60 CFU in 100 μl PBS), or s.c. (5 × 10^2^–5 × 10^5^ CFU in 50 μl PBS). *L. longbeachae* NSW150 was delivered by i.n. instillation (10^4^ CFU in 50 μl PBS). Detailed protocols for inoculum preparation and i.n. instillation were described elsewhere^58^. To block MR1-mediated responses, in some experiments mice were injected i.p. four times with 0.25 mg anti-MR1 (8F2.F9^59,60^) or isotype control (3E12^61^) antibodies (prepared in-house) on days −1, 1, 3, and 5 (relative to infection on day 0). Mice were weighed daily and assessed for visual signs of clinical disease, including inactivity, ruffled fur, labored breathing, and huddling behavior. Animals that had lost ≥20% of their original body weight and/or displayed evidence of pneumonia were killed.

### Determination of bacterial counts in infected organs

Bacterial colonization was determined by counting CFU obtained from plating homogenized organs in duplicate or 100 μl whole blood, from infected mice (5 per group) on CHA (for *F. tularensis*) or BYE (for *L. longbeachae*) agar containing the appropriate antibiotics (listed above) and colonies counted after 4 days at 37 °C under aerobic conditions.

### Vaccination

Age- and gender-matched C57BL/6 and *Mr1*^−/−^ mice were inoculated (i.v.) with 10 nmol CpG together with 5-OP-RU (2 nmol in 200 μl) on day 0, followed by three additional 5-OP-RU injections at day 1, 3, and 5. Control mouse groups included untreated C57BL/6 and CpG alone vaccinated. Immunized mice were left for 4 weeks prior to challenge with either *F. tularensis* LVS or *L. longbeachae*.

### Preparation of cells for flow cytometry

Detailed protocols for preparation of samples for flow cytometry from various organs and blood, were described elsewhere^58^. Briefly, for lungs, perfusion through the heart was performed with 10 ml cold PBS and lung single-cell suspensions were prepared by finely chopping the lungs, followed by collagenase (type IV) digestion and red blood cell (RBC) lysis. Liver MAIT cells were prepared after by portal vein perfusion with 10 ml cold PBS and pushing the liver tissue through 70 μm cell strainers, followed by gradient (33.75%:70% Percoll) centrifugation to enrich lymphocytes and RBC lysis. Samples for which there was poor perfusion were excluded in an unbiased manner prior to staining. For blood, MAIT cells were analyzed from 200 μl whole blood after RBC lysis. IELs and LPLs were prepared essentially as previously described^62,63^. Briefly, the SI was collected into Hank’s buffered salt solution (HBSS) containing 2% FCS after perfusion through the heart with 10 ml cold PBS. Peyer’s patches and fat were removed by dissection, the SI cut longitudinally, then into 2 cm pieces, and vortexed vigorously for 30 s. IELs were dislodged from the SI tissue with vigorous shaking (230 r.p.m.) for 30 min at 37 °C in 30 ml HBSS buffer supplemented with HEPES, 8% FCS, and 1 mM EDTA. IEL containing supernatant was then filtered through 70 μm cell strainers. The remaining tissue was then digested with collagenase type I (Worthington, 100 U/ml; 35 ml/mouse) at 37 °C for 45 min with shaking (230 r.p.m.). The digest, containing LPLs, was pushed through a 70 μm cell strainer. Both IELs and LPLs were separated from epithelial cells via Percoll gradient (44%:70%) centrifugation and kept on ice prior to staining for flow cytometry. The absolute numbers of MAIT and non-MAIT T cells were calculated with reference to the number of counting beads (Sphero Rainbow Calibration Particles, BD Biosciences) added to each sample prior to flow cytometry.

### MAIT cell preparation for cell sorting and adoptive transfer

As MAIT cell frequencies are low in naive mice, prior to adoptive transfer experiments, MAIT cell populations were expanded in WT (C57BL/6) or cytokine knockout mice by vaccination (as above) to increase MAIT cell numbers. After 7 days, single cells were prepared from the liver live CD3^+^CD45^+^MR1-5-OP-RU tetramer^+^ cells sorted using a BD FACS Aria III. Detailed protocols for the preparation of samples for fluorescence-activated cell sorting (FACS) are described elsewhere^58^. MAIT cells (10^5^) were injected into the tail veins of recipient mice, which then received 0.1 mg each of anti-CD4 (GK1.5) and anti-CD8 (53.762) mAb i.p. on days 2 and either 5 or 6, to control residual conventional T cells. Mice were rested for 2 weeks post transfer, to allow full expansion of the MAIT cell population prior to subsequent infectious challenge.

#### Antibodies and flow cytometry

Antibodies against murine CD45.2 (Clone 104, Cat #553772, fluorescein isothiocyanate, 1:200), CD19 (clone 1D3, Cat #551001, PerCPcy5.5, 1:200), TCRβ (clone H57-597, Cat #553174, APC, 1:200 or Cat #553172, PE, 1:200), CD44 (clone IM7, Cat #612799, BUV737, 1:200 or Cat #740215, BUV395, 1:200), CD4 (clone GK1.5, Cat #552051, APC-Cy7, 1:200), IFNγ (clone XMG1.2, Cat #557649, PE-Cy7, 1:400), IL-17A (clone TC11-18H10, Cat #559502, PE, 1:200), GM-CSF (clone MP1-22E9, Cat #554406, PE, 1:200), TNF (clone MP6-XT22, Cat #557644, PE-Cy7, 1:200), Ly6G (clone 1A8, Cat #560601, PEcy7, 1:200), CD103 (clone M290, Cat #740238, BUV395, 1:200), CD11b (clone M1/70, Cat #557657, APC-Cy7, 1:200), Ly6C (clone AL-21, Cat #563011, BV605, 1:200), CD49a (clone Hα31/8, Cat #740375, BV605, 1:200), CD62L (clone MEL-14, Cat #565159, APC-R700, 1:400), and CD8β (clone:H35-17.2, Cat#747505, BV750, 1:400) were purchased from BD Biosciences (Franklin Lakes, NJ). Antibodies against CD8a (clone 53-6.7, #12-0081-83, PE, 1:1000), RORγt (clone: B2D, #17-6981-82, APC, 1 : 200), T-bet (clone: 4B10, #25-5825-82, PE-Cy7, 1:200), CD49b (clone DX5, #25-5971-81, PE-Cy7, 1:200), IL-18Ra (clone P3TUNYA, #48-5183-82, eFluor 450, 1:400), CD69 (clone H1.2F3, #15-0691-82, PE-Cy5, 1:200), and CD45 (clone: 30-F11, #58-0451-82, AF532, 1:200) were purchased from eBioscience (San Diego, CA). Abs against F4/80 (clone BM8, Cat #123116, APC, 1:200), MHCII (clone M5/114, Cat #107631, BV421, 1:200), CD11c (clone N418, Cat #117308, PE, 1:200), NK1.1 (clone PK136, Cat #108731, BV421, 1:200) and CD127 (clone A7R34, Cat #135031, PE/Dazzle 594, 1:200), TCRβ (clone:H57-597, Cat#109246, APC/Fire 750, 1:400), and CD8α (clone:53-6.8, Cat#100782, Spark NIR685, 1:400) were purchased from BioLegend (San Diego, CA). Blocking Ab (26.5: anti-human MR1 MoAb or 8F2.F9: anti-mouse MR1 MoAb) and isotype controls (3E12, 8A5), as well as MR1 tetramers, were prepared in-house.

To block nonspecific staining, cells were incubated with unlabeled MR1-6-FP tetramer and anti-Fc receptor (2.4G2) for 15 min at room temperature and then incubated at room temperature with Ab/tetramer cocktails in PBS/2% FCS. 7AAD (Sigma-Aldrich) was added during antibody staining.

Cells were fixed with 1% paraformaldehyde prior to analysis on LSRII or LSR Fortessa or Canto II (BD Biosciences) or Aurora (Cytek) flow cytometers. For intracellular cytokine staining, Golgi plug (BD Biosciences) was used during all processing steps. Cells stimulated with PMA/ionomycin (20 ng/ml and 1 μg/ml, respectively) for 3 h at 37 °C were included as positive controls. Fixable Viability Dye (eBioscience) was added for 30 min at 4 °C before surface staining. Surface staining was performed at room temperature and cells were stained for intracellular cytokines using the BD Fixation/Permeabilization Kit (BD, Franklin Lakes, NJ) or TFs using the transcription buffer staining set (eBioscience) according to the manufacturers’ instructions.

#### scRNA-seq and analysis

MAIT cells were sorted (BD FACS Aria III) from the pooled livers of 17 naive C57BL/6 mice or C57BL/6 mice infected with 10^4^ CFU *F. tularensis* LVS for 13 (pooled from 2 mice) or 48 days (pooled from 4 mice). Approximately 3500 MAIT cells from naive mice or 10,000 at 13 and 48 dpi were loaded per channel onto a Chromium controller (10× Genomics) for generation of gel bead-in-emulsions using 3′-gene expression kits (v3; 10× Genomics). cDNA libraries were generated according to the manufacturer’s instructions (10× Genomics) and then converted using the MGIEasy Universal Library Conversion Kit (BGI) before sequencing on a MGISEQ-2000 instrument (BGI). FASTQ files were processed using the cellranger count pipeline from Cell Ranger version 3.1.0 (10× Genomics) with 10× mouse genome 2020-A release as a reference. Only genes expressed in three or more cells were considered. Cells outside the threshold of 200–4000 expressed genes and those with more than 15% mitochondrial content were excluded. scRNA-seq data were normalized and highly variable genes identified using the SCTransform function from Seurat version 3.1.5. PCA was performed using the Seurat’s RunPCA function. The PCA output was used to generate UMAP using RunUMAP function from Seurat, with 30 principal components as input. Unsupervised cell clustering was performed using the Seurat’s FindClusters function, with resolution parameter of 0.7. DGEA was performed using the Seurat’s FindMarkers function at default parameters, by comparing between clusters as specified in each analysis.

### Statistical analysis

Statistical tests were performed using the Prism GraphPad software (version 9.1 La Jolla, CA). Comparisons between groups were performed using Student’s *t*-tests or analysis of variance tests as appropriate, unless otherwise stated. Survival curves were compared using the Log-rank (Mantel–Cox) test for multiple groups. Flow cytometric data analysis was performed with FlowJo10 software (Ashland, OR).

### Reporting summary

Further information on research design is available in the Nature Research Reporting Summary linked to this article.

## Supplementary information

Supplementary information

Reporting summary

## Data Availability

All relevant data from this study, including raw flow cytometry data, are available from the corresponding authors upon reasonable request. The full raw single-cell RNA-sequencing data generated has been submitted to ArrayExpress database (10× Genomics scRNA-seq data under accession number E-MTAB-10239). Source data are provided with this paper.

## Source data

Source Data
