# Supplementary material for: Francisella tularensis induces Th1 like MAIT cells conferring protection against systemic and local infection
Source: Nat Commun. 2021 Jul 16;12:4355. doi: 10.1038/s41467-021-24570-2 (PMC8285429; doi:10.1038/s41467-021-24570-2)
Supplement: Supplementary file 1 — Supplementary information [file 41467_2021_24570_MOESM1_ESM.pdf]

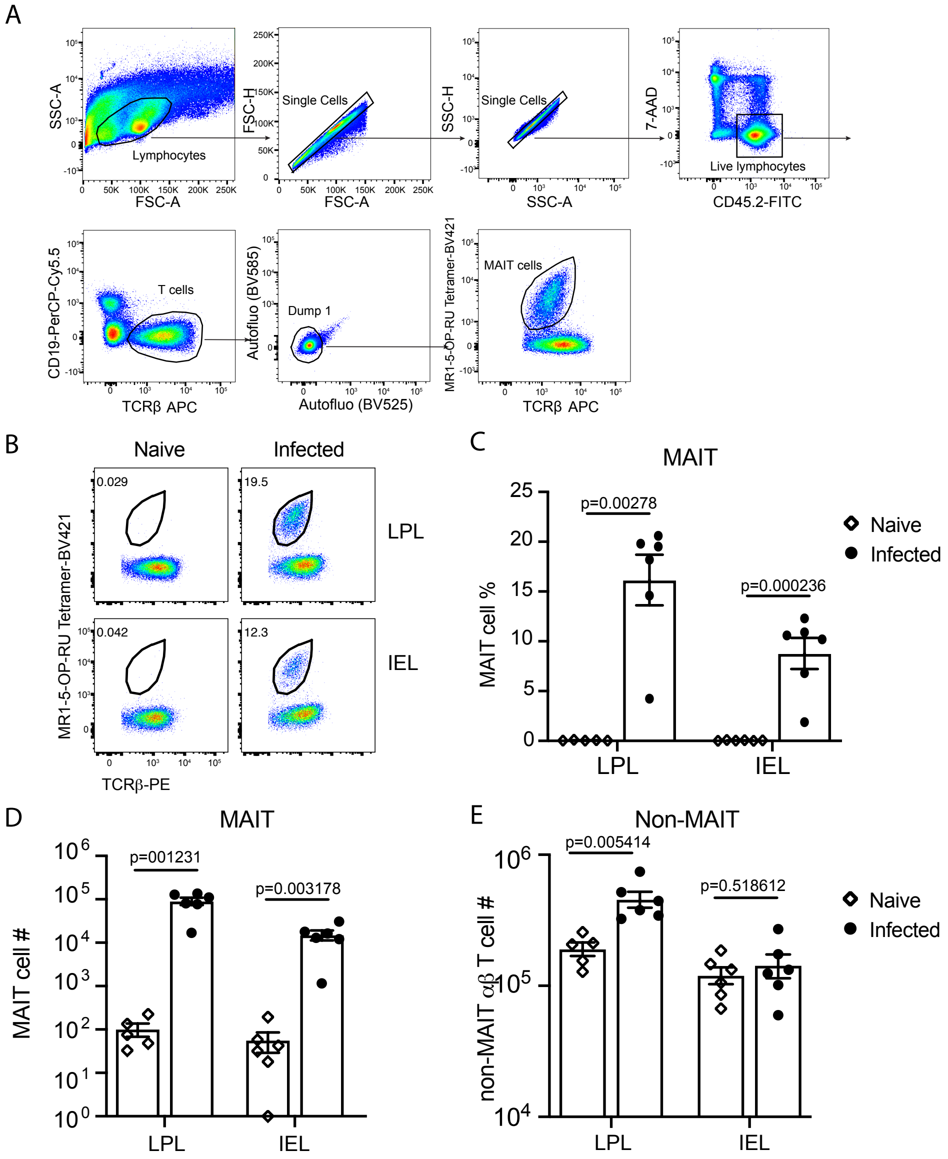

Supplementary Figure 1. Gating strategy for flow cytometry analysis of murine MAIT cells and intestinal MAIT cell analysis. Murine MAIT cells are identified by gating on the lymphocyte population then excluding doublets using forward scatter and side scatter properties; 7AAD$^-$CD45$^+$ live lymphocytes are gated and further selected as CD19$^-$TCRβ$^+$; auto-fluorescent cells are excluded using fluorescence in BV525 and BV585 channels (which flank the BV421 channel used for MR1:5-OP-RU tetramer) and TCRβ$^+$ T cells or TCRβ$^+$, MR1:5-OP-RU tetramer$^+$ MAIT cells are then gated as shown. This gating was used for all experiments except for those shown in Supplementary Figures 6 and 10. (B) Representative FACS plots and (C) percentage of MAIT cells among TCRβ$^+$ lymphocytes. (D) absolute numbers of MAIT cells and (E) non-MAIT T cells, in the small intestine IEL and LPL of C57BL/6 mice either uninfected or intravenously infected with 10^4 CFU *F. tularensis* LVS for 6 days. Data represent mean ± SEM. n=5 (naïve LPL) or 6 (all other samples) mice examined over 2 independent experiments. Multiple unpaired t-test (two-tailed). P values are indicated. ns: non-significant (Related to Fig. 1). Source data are provided as a Source Data file.

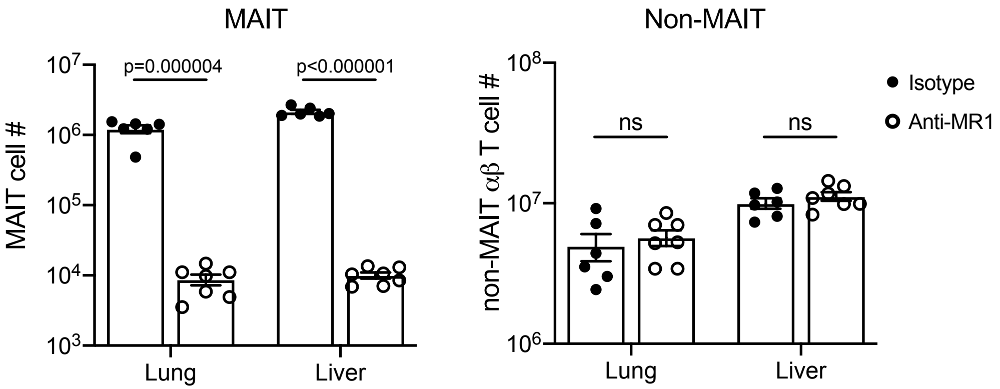

Supplementary Figure 2. MAIT cell accumulation during *F. tularensis* infection is MR1-dependent. WT (C57BL/6) mice were infected i.v. with 10^4 CFU *F. tularensis* LVS on day 0 and were injected i.p. four times with 0.25 mg anti-MR1 (8F2.F9) or isotype (3E12) antibodies on days -1, 1, 3, and 5. Absolute numbers of MAIT cells and non-MAIT αβ-T cells were assessed in the lung and liver at 6 dpi. Pooled data, mean ± SEM of n=6 (isotype) or n=7 (anti-MR1) mice per group from 2 independent experiments with similar results. Multiple unpaired t-test, p values are indicated. ns; non-significant (P>0.05). (Related to Fig. 1). Source data are provided as a Source Data file.

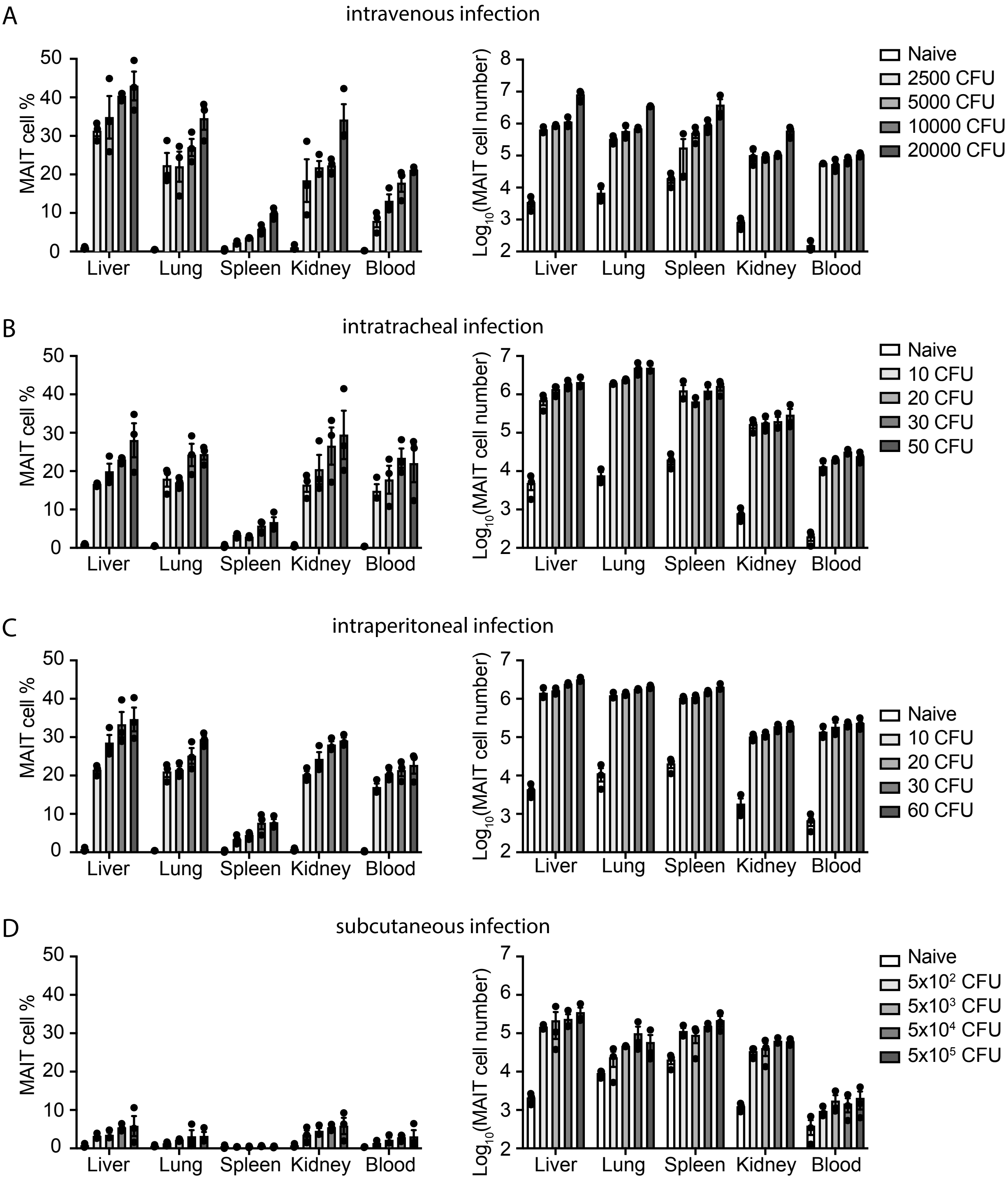
A
intravenous infection
MAIT cell %
Log10(MAIT cell number)
Liver
Lung
Spleen
Kidney
Blood
Naive
2500 CFU
5000 CFU
10000 CFU
20000 CFU
B
intratracheal infection
10 CFU
20 CFU
30 CFU
50 CFU
C
intraperitoneal infection
60 CFU
D
subcutaneous infection
5x10^2 CFU
5x10^3 CFU
5x10^4 CFU
5x10^5 CFU

Supplementary Figure 3. Dose dependent expansion of MAIT cells *in vivo* by *F. tularensis* infection is similar via different infection routes. (A-D) Relative and absolute number (mean ± SEM) of MAIT cells in liver, lungs, spleen, kidney and blood (200 µl) from uninfected or infected mice with indicated doses of *F. tularensis* LVS at 14 dpi, (A) intravenously, (B) intratracheally, (C) intraperitoneally and (D) subcutaneously. Data show mean ± SEM and individual mice; n=3 mice. Experiment was repeated once with similar results. (Related to Fig. 1). Source data are provided as a Source Data file.

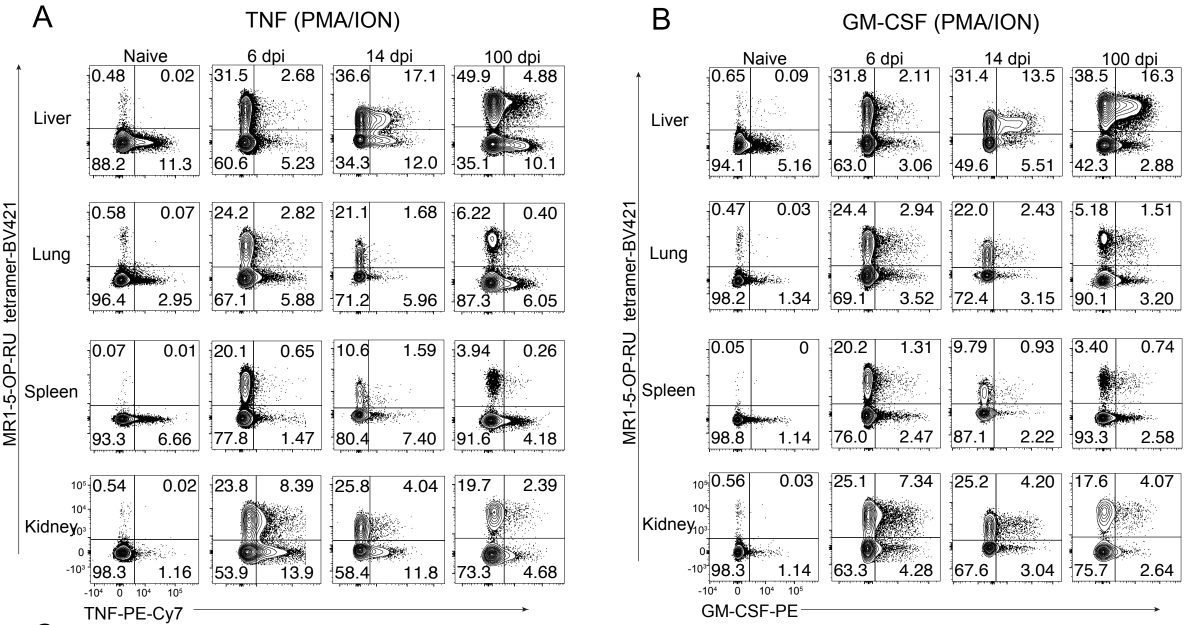

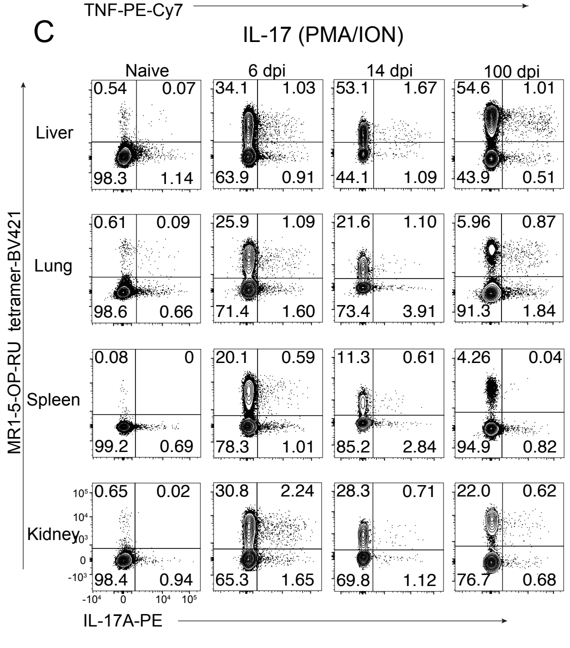

Supplementary Figure 4. Representative flow cytometry plots showing intracellular cytokine staining for (A) TNF, (B) GM-CSF and (C) IL-17 of TCRβ$^+$ lymphocytes (including MAIT cells: MR1:5-OP-RU tetramer$^+$ and non-MAIT T cells: MR1:5-OP-RU tetramer$^-$) after 4 h of culture with brefeldin A and PMA/ionomycin stimulation from liver, lungs, spleen and kidneys of naïve mice and infected mice on 6, 14 and 100 dpi with 2×10^4 CFU *F. tularensis* LVS i.v. (Related to Fig. 3).

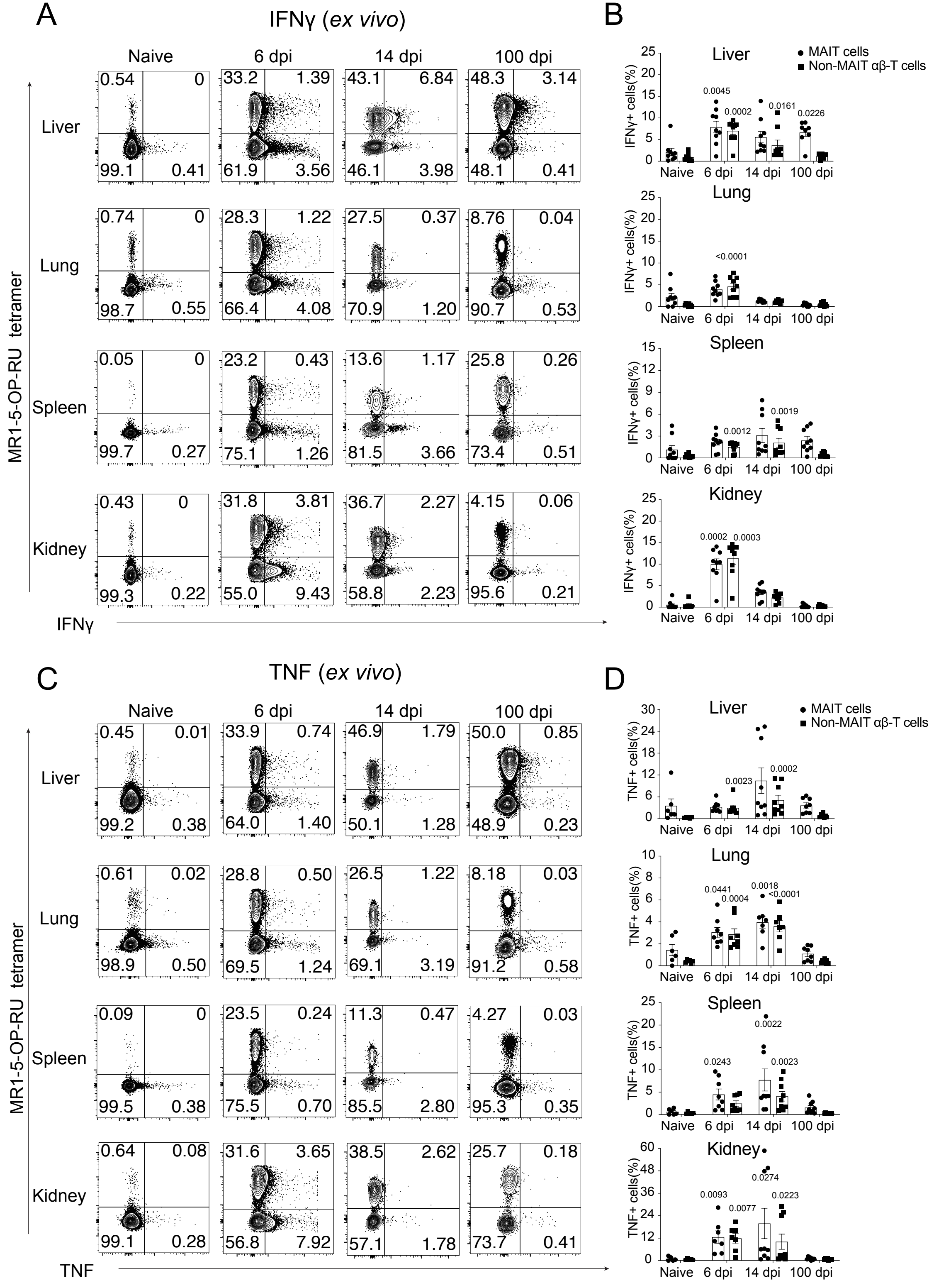

A
IFNγ (ex vivo)
Naive
6 dpi
14 dpi
100 dpi
Liver
Lung
Spleen
Kidney
MR1-5-OP-RU tetramer
IFNγ
0.54 0 99.1 0.41
33.2 1.39 61.9 3.56
43.1 6.84 46.1 3.98
48.3 3.14 48.1 0.41
0.74 0 98.7 0.55
28.3 1.22 66.4 4.08
27.5 0.37 70.9 1.20
8.76 0.04 90.7 0.53
0.05 0 99.7 0.27
23.2 0.43 75.1 1.26
13.6 1.17 81.5 3.66
25.8 0.26 73.4 0.51
0.43 0 99.3 0.22
31.8 3.81 55.0 9.43
36.7 2.27 58.8 2.23
4.15 0.06 95.6 0.21
B
MAIT cells
Non-MAIT αβ-T cells
IFNγ+ cells(%)
Liver
0.0045
0.0002
0.0161
0.0226
Lung
<0.0001
Spleen
0.0012
0.0019
Kidney
0.0002
0.0003
C
TNF (ex vivo)
TNF
0.45 0.01 99.2 0.38
33.9 0.74 64.0 1.40
46.9 1.79 50.1 1.28
50.0 0.85 48.9 0.23
0.61 0.02 98.9 0.50
28.8 0.50 69.5 1.24
26.5 1.22 69.1 3.19
8.18 0.03 91.2 0.58
0.09 0 99.5 0.38
23.5 0.24 75.5 0.70
11.3 0.47 85.5 2.80
4.27 0.03 95.3 0.35
0.64 0.08 99.1 0.28
31.6 3.65 56.8 7.92
38.5 2.62 57.1 1.78
25.7 0.18 73.7 0.41
D
TNF+ cells(%)
0.0023
0.0002
0.0441
0.0004
0.0018
<0.0001
0.0243
0.0022
0.0023
0.0093
0.0077
0.0274
0.0223

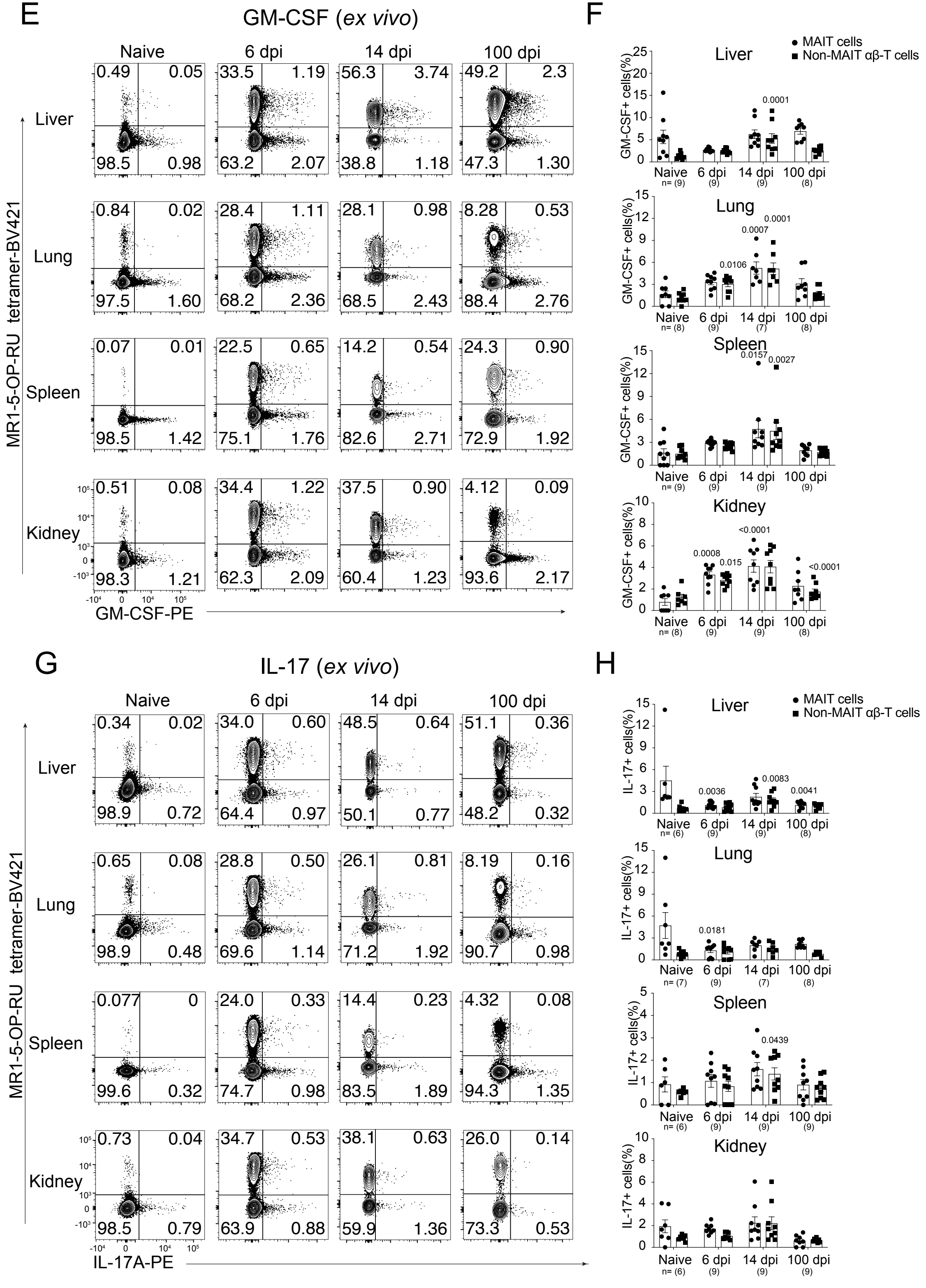
E
GM-CSF (ex vivo)
Naive
6 dpi
14 dpi
100 dpi
Liver
Lung
Spleen
Kidney
MR1-5-OP-RU tetramer-BV421
GM-CSF-PE
F
MAIT cells
Non-MAIT αβ-T cells
Liver
Lung
Spleen
Kidney
GM-CSF+ cells(%)
n= (9)
0.0001
0.0007
0.0106
0.0157
0.0027
<0.0001
0.0008
0.015
G
IL-17 (ex vivo)
IL-17A-PE
H
IL-17+ cells(%)
0.0036
0.0083
0.0041
0.0181
0.0439

Supplementary Figure 5. Cytokine production detected directly *ex vivo*, following *F. tularensis* infection. Representative flow cytometry plots (A, C, E, G) and cell percentage (B, D, F, H) showing intracellular cytokine staining for (A, B) IFNγ, (C, D) TNF, (E, F) GM-CSF and (G, H) IL-17 detected directly *ex vivo* (without *in vitro* stimulation) in TCRβ^{+} lymphocytes (including MAIT cells: MR1:5-OP-RU tetramer^{+} and non-MAIT T cells: MR1-5-OP-RU tetramer^{-}) after 4 h of culture with brefeldin A from liver, lungs, spleen and kidneys of uninfected mice and infected mice on 6, 14 and 100 dpi with 2×10^{4} CFU *F. tularensis* LVS i.v. Pooled data from 2 independent experiments with similar results (mean ± SEM) n=6-9 mice, as indicated. One-way ANOVA comparing percentages of cytokine producing MAIT cells or non-MAIT αβ-T cells at indicated time points post infection with the matching uninfected groups, P values <0.05 are indicated (Related to Fig. 3). Source data are provided as a Source Data file.

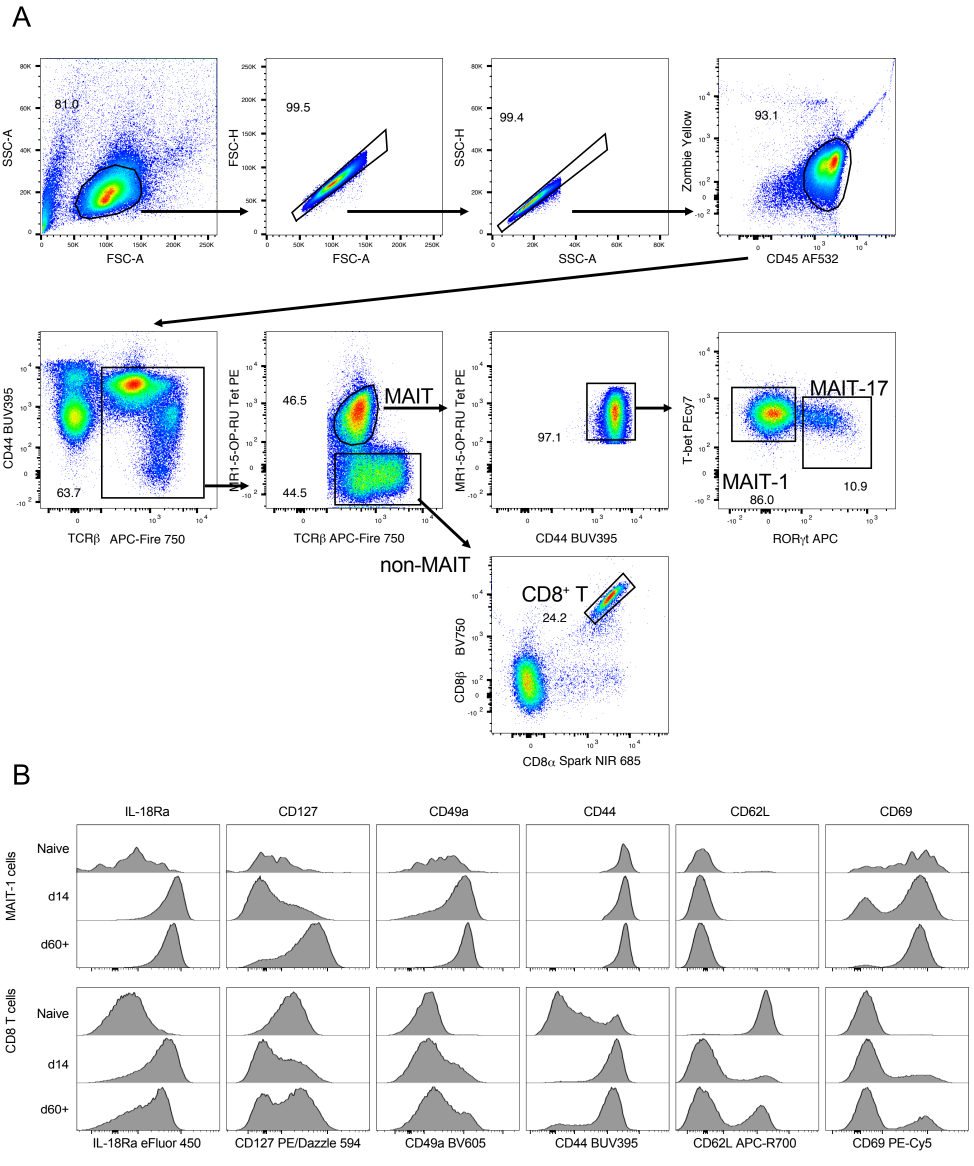

Supplementary Figure 6. Expression of memory markers by MAIT cells during the course of *F. tularensis* infection. A. Gating strategy for MAIT-1 and $CD8^+$ non-MAIT T cells analysed in (B). B. WT (C57BL/6) mice were infected i.v. with 10^4 CFU *F. tularensis* LVS. Representative FACS plots showing surface expression (one mouse per group) of IL-18R, CD127, CD49a, CD44, CD62L and CD69 on liver MAIT1 cells, or CD8 T non-MAIT T cells at indicated time points. One representative experiment is shown, from 2 experiments performed on independent occasions with similar results (n=5 for naïve or 60+ dpi (61 and 63 dpi in two experiments), n=3 for 14 dpi). (Related to Fig. 1).

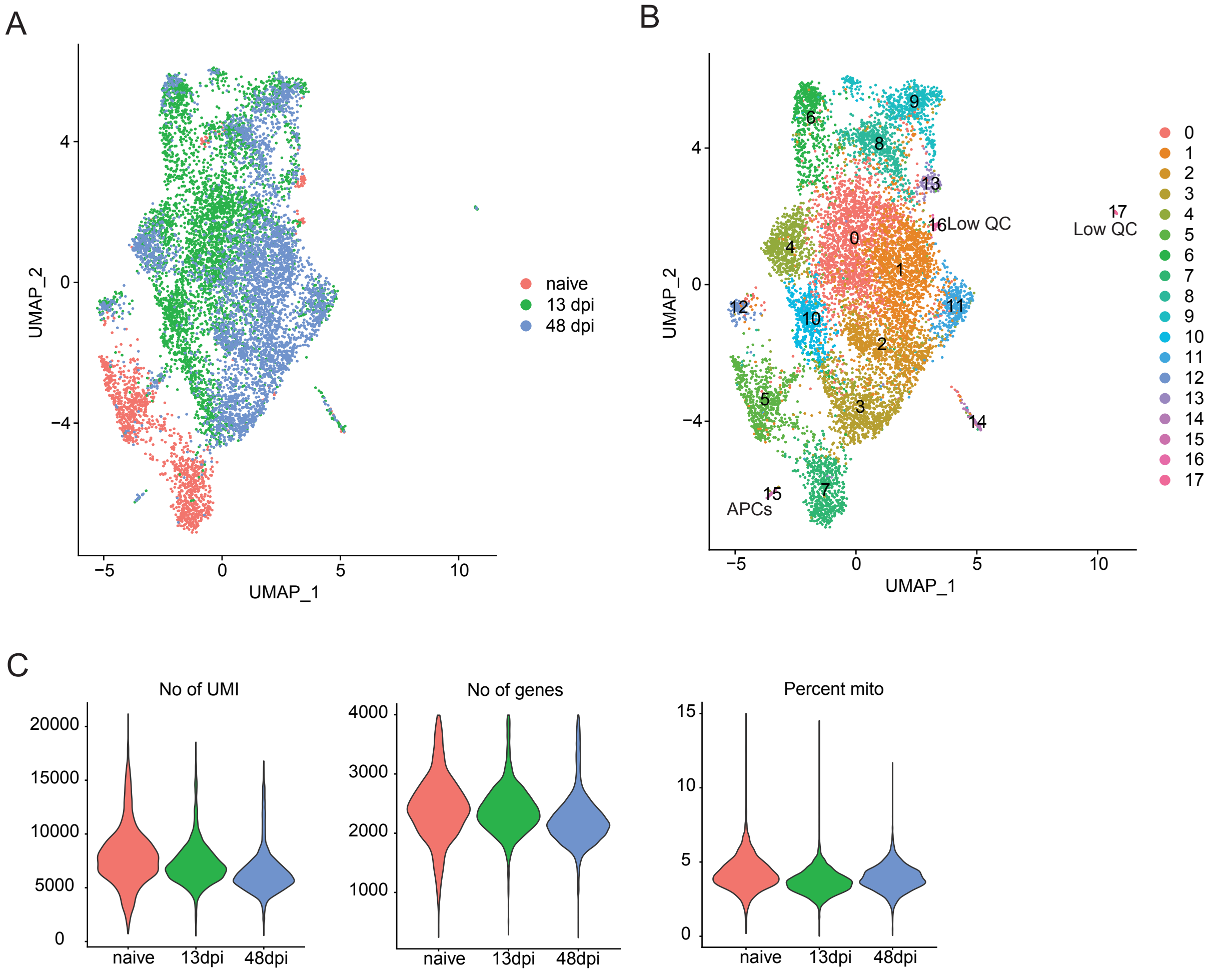

Supplementary Figure 7. Clustering and quality control metrics for scRNA-seq of liver MAIT cells before and upon *F. tularensis* LVS infection. (A) UMAP representation of liver MAIT cells from naïve mice and mice at days 13 and 48 post infection prior to removal of clusters showing low quality control metrics, calculated using principal components (PC) 1–30. (B) Unsupervised clustering on a UMAP representation of liver MAIT cells. (C) Violin plots of the number of unique molecular identifier (UMI) transcripts, numbers of genes detected per cell, and percentage of mitochondrial genes detected per cell, with a filter of 200-4,000 expressed genes and less than 15% mitochondrial content).

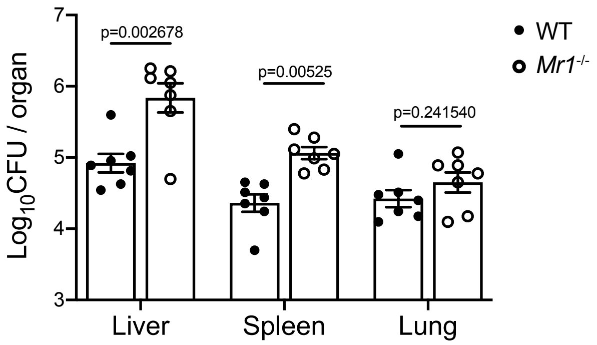

Supplementary Figure 8. ***Mr1***$^{-/-}$ **mice that succumbed to infection had high bacterial loads.** The bacterial burden (CFU) in the liver, spleen and lung was assessed at 7 dpi in *Mr1*$^{-/-}$ mice reaching humane endpoints from Fig. 6F, and paired WT mice. Pooled data (mean ± SEM, n = 7 mice per group) from 3 independent experiments with similar results, multiple unpaired t-test, p values are indicated. (Related to Fig. 6). Source data are provided as a Source Data file.

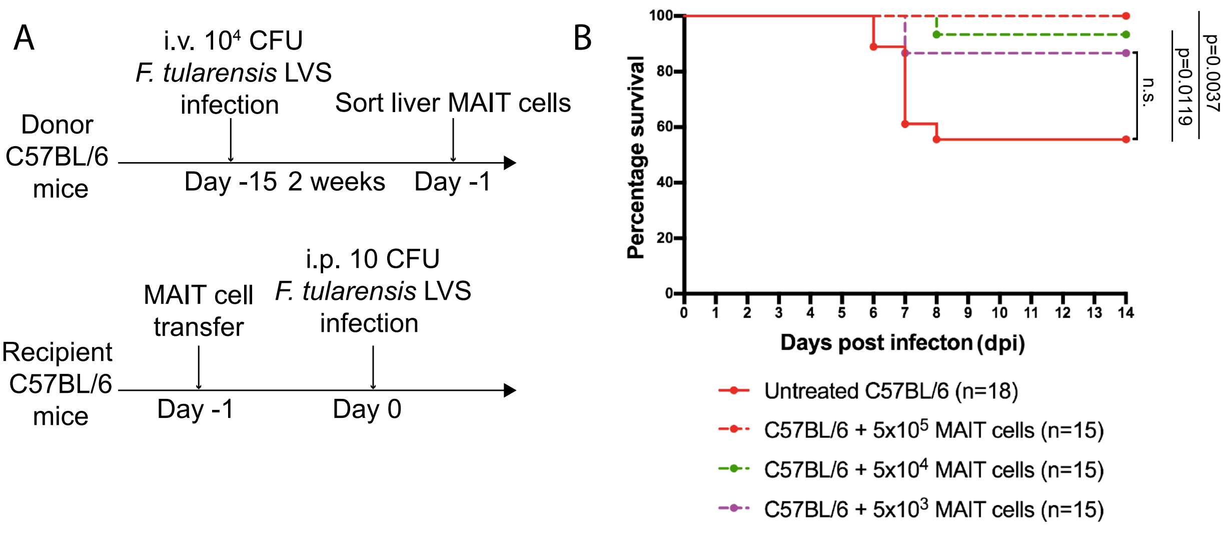

Supplementary Figure 9. Adoptive transfer of MAIT cells rescues WT C57BL/6 mice from fatal *F. tularensis* peritonitis in a dose dependent manner. (A) Schematic flow charts of enriching and sorting MAIT cells from donor wildtype C57BL/6 mice (upper chart). These cells were then adoptively transferred (i.p.) to recipient C57BL/6 mice (numbers indicated in B), followed by challenge with a lethal dose (10 CFU) of *F. tularensis* LVS (i.p.). (B) Survival of C57BL/6 mice untreated or receiving $5x10^3$, $5x10^4$, or $5x10^5$ MAIT cells, after challenge with *F. tularensis* LVS. Log-rank test. n.s.: non-significant. (Related to Fig. 6 and 7). Source data are provided as a Source Data file.

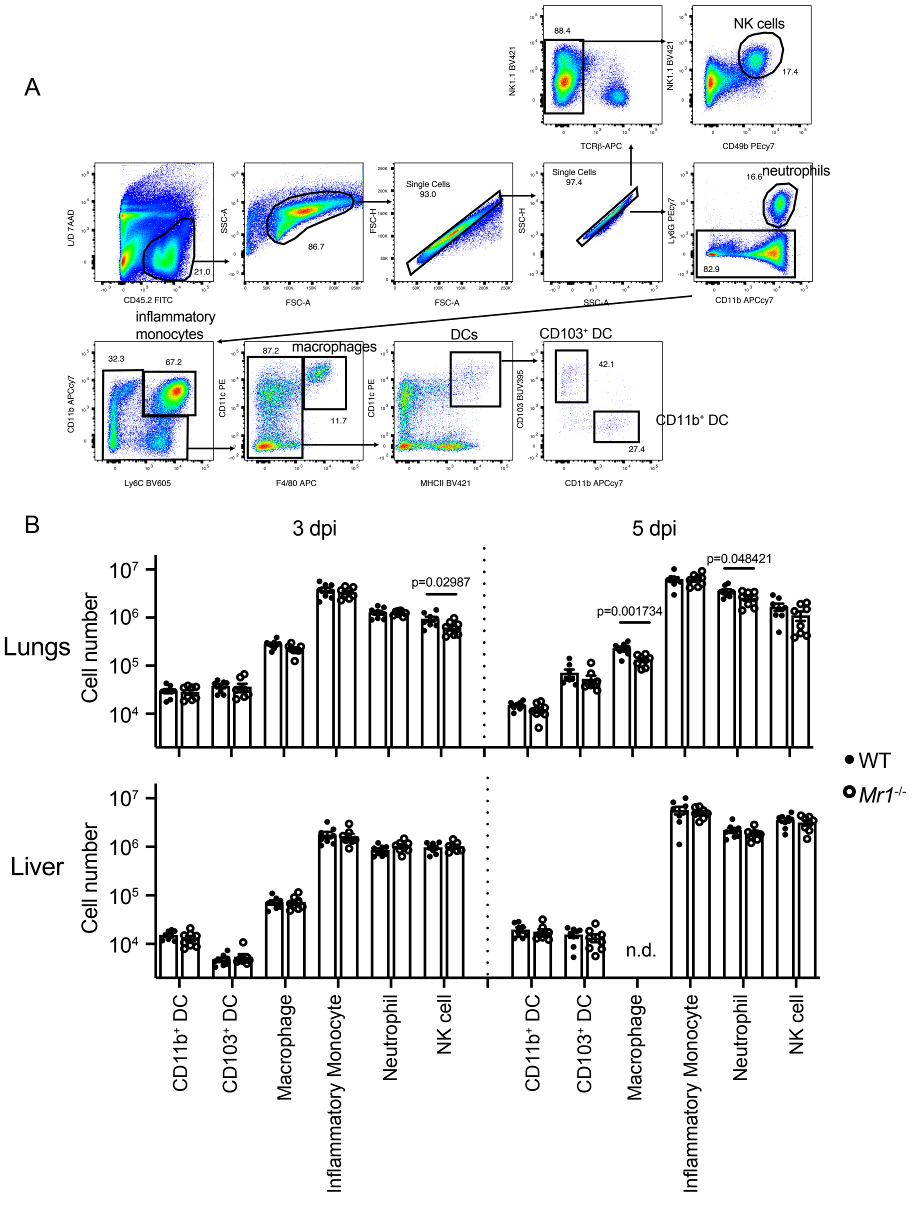

Supplementary Figure 10. Innate cell responses to infection are minimally altered in *Mr1*$^{-/-}$ mice. (A) gating strategy. WT (C57BL/6) or *Mr1*$^{-/-}$ mice were infected i.v. with 10^4 CFU *F. tularensis* LVS. (B) Absolute numbers of innate immune cells (neutrophils (Ly6G$^+$CD11b$^+$), NK cells (NK1.1$^+$CD49b$^+$TCRβ$^-$), macrophages (CD11c$^+$F4/80$^+$), CD11b$^+$ and CD103$^+$ DCs (MHCII^{high-}CD11c^{high}), from the lung and liver (as indicated) were assessed at 3 and 5 dpi. n.d., not determined. Macrophages could not be accurately enumerated in the livers at day 5 due to their low numbers. Pooled data (mean ± SEM) from 2 independent experiments with similar results using 8 mice per group at each time point. Multiple unpaired t-test (two-tailed), p values are indicated (all others were non-significant; $p>0.05$). (Related to Fig. 6 and 7). Source data are provided as a Source Data file.

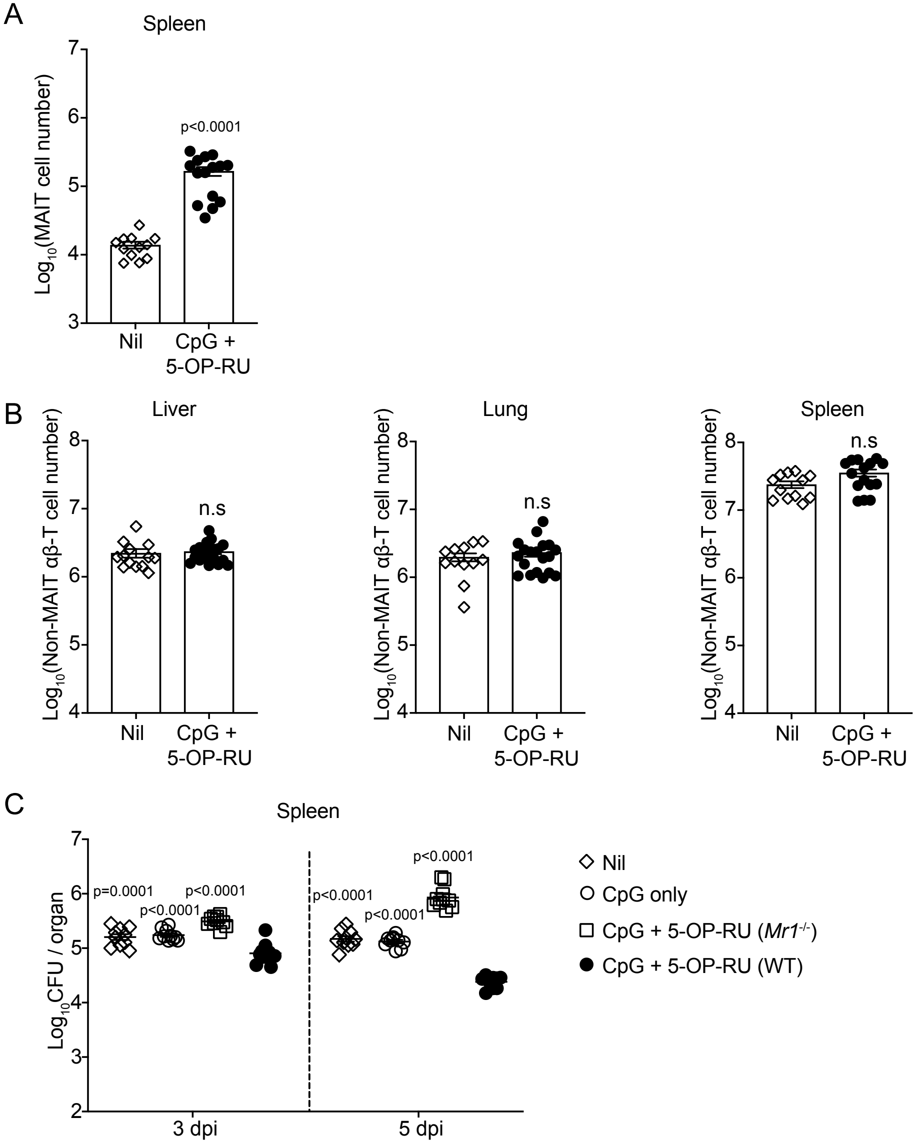

Supplementary Figure 11. Vaccination with 5-OP-RU and CpG specifically boosts MAIT cell numbers and confers systemic protection. Mice were immunised as depicted in Figure 8. (A) Total MAIT cells in the spleen from naïve (n=12) and vaccinated (n=15) mice (treated with CpG + 5-OP-RU), and (B) non-MAIT αβ-T cells in the lung (n=12 naïve, n=19 vaccinated), liver (n=13 naïve, n=19 vaccinated) and spleen (n=12 naïve, n=15 vaccinated). Pooled data from 3 independent experiments with similar results (mean ± SEM). Unpaired t-test (two-tailed), n.s.: non-significant. (C) Bacterial burden was assayed in the spleen from unvaccinated WT mice (open diamond), mock vaccinated WT mice (CpG only, open circle), vaccinated *Mr1*$^{-/-}$ mice (CpG + 5-OP-RU, open square) and vaccinated WT mice (CPG + 5-OP-RU, filled circle) at indicated time points post *F. tularensis* infection (2.5×10^4 CFU, i.v.). Pooled data from 3 independent experiments with similar results (mean ± SEM, n=10 mice per group). One-way ANOVA on log-transformed pooled data (Related to Fig. 8). Source data are provided as a Source Data file.

Supplementary Table 1

List of differentially expressed genes between MAIT-1 (cluster 5) and MAIT-17 (cluster 7) cells from naïve mice, related to Figure 5C.
Statistical analysis was performed using two-sided wilcoxon rank sum test implemented in Seurat v3 FindMarkers() function.
Positive log fold-change: Upregulated in Cluster 5; Negative log fold-change: Downregulated in Cluster 5 Negative log fold-change: Downregulated in Cluster 5
(avg_logFC: average log-transformed fold-change, p.value: raw p-value, p_val_adj: p-value adjusted for multiple comparisons (bonferroni correction))

	p_val	avg_logFC	p_val_adj
Ccl5	6.52E-117	2.29217361	9.81E-113
Ly6c2	3.52E-63	1.285127976	5.29E-59
Ms4a4b	3.23E-139	1.172207962	4.85E-135
Xcl1	2.64E-108	1.091584461	3.97E-104
Gimap7	3.39E-134	1.086902791	5.10E-130
Gzmb	1.04E-56	1.042652627	1.56E-52
Klrd1	7.58E-86	1.038001586	1.14E-81
Nkg7	2.34E-107	0.961273727	3.52E-103
Il2rb	7.56E-100	0.95115831	1.14E-95
Ifi27l2a	8.66E-63	0.930124488	1.30E-58
Klrc1	1.51E-61	0.92506294	2.27E-57
Bcl2	1.31E-81	0.862241388	1.97E-77
Klre1	1.46E-48	0.851887409	2.20E-44
Gimap4	2.01E-105	0.84790221	3.02E-101
Klrc2	5.08E-62	0.846365136	7.63E-58
Inpp4b	6.71E-85	0.815690736	1.01E-80
Ms4a6b	4.97E-94	0.791122216	7.47E-90
Cd160	5.80E-64	0.738771265	8.73E-60
Hopx	1.41E-95	0.738344007	2.12E-91
Gm2682	4.04E-87	0.733488161	6.08E-83
H2-Q7	1.59E-81	0.713695581	2.40E-77
Plac8	6.92E-49	0.701605287	1.04E-44
AW112010	3.37E-70	0.677674756	5.07E-66
Gramd3	9.80E-77	0.632703756	1.47E-72
Epsti1	1.43E-59	0.619968881	2.15E-55
Art2b	2.24E-76	0.615108587	3.37E-72
Gimap3	1.49E-68	0.613840073	2.24E-64
Lyst	1.64E-44	0.603091825	2.47E-40
Gimap6	2.64E-69	0.598023282	3.97E-65
Ctla2a	2.44E-48	0.585133322	3.67E-44
Ctsw	1.43E-67	0.584865934	2.15E-63
Rgs1	1.09E-33	0.578700733	1.64E-29
Dusp2	3.76E-35	0.570369606	5.66E-31
Ifng	4.08E-43	0.547227	6.13E-39
Rcbtb2	4.23E-23	0.521152077	6.36E-19
Fyb	6.65E-47	0.519371454	1.00E-42
Gm19585	1.21E-40	0.515800636	1.83E-36
Klrb1c	6.07E-48	0.513666359	9.13E-44
Gpr174	2.34E-56	0.492800372	3.52E-52
Cd6	2.06E-44	0.492027533	3.10E-40
Klrk1	4.89E-15	0.488822135	7.36E-11
Itga1	1.42E-41	0.471800823	2.14E-37
Satb1	2.11E-38	0.452858919	3.17E-34
H2-K1	7.82E-65	0.452507782	1.18E-60
Mbnl1	5.17E-45	0.450605413	7.78E-41
Ugcg	8.46E-34	0.447932612	1.27E-29
Ptprc	2.35E-43	0.436552385	3.53E-39
Malt1	1.56E-31	0.430725759	2.34E-27
Slamf7	1.62E-40	0.430263301	2.43E-36
Sh3bgrl3	1.52E-50	0.425542864	2.28E-46
Itgb2	4.44E-36	0.420280629	6.68E-32
Samd3	7.16E-45	0.418562116	1.08E-40
Il21r	1.85E-47	0.413071145	2.78E-43
Zfp36l2	2.06E-25	0.412149063	3.10E-21
Ctsd	2.28E-34	0.405303352	3.43E-30
Cxcr3	3.00E-36	0.391980376	4.51E-32
B4galnt1	7.29E-30	0.38948441	1.10E-25
Peli1	1.83E-26	0.380727285	2.75E-22
Rac2	4.86E-49	0.375576498	7.31E-45
Klra9	1.12E-16	0.374939301	1.68E-12
Dennd4a	4.30E-18	0.359170386	6.46E-14
Fasl	1.37E-30	0.353491242	2.06E-26
Atp8a2	3.00E-33	0.349545048	4.51E-29
Ptpn18	8.75E-42	0.348742087	1.32E-37
Cd52	6.41E-42	0.3457739	9.64E-38
Ptpn22	9.61E-25	0.338844739	1.44E-20
Ly6a	1.20E-19	0.337384468	1.81E-15
Sp100	6.88E-24	0.331136942	1.03E-19
B2m	2.93E-49	0.330378244	4.41E-45
BE692007	9.05E-29	0.327596766	1.36E-24
Efhd2	2.78E-22	0.320537746	4.17E-18
Chsy1	2.56E-27	0.319777692	3.85E-23
Pde3b	7.54E-18	0.317741524	1.13E-13
Gimap5	4.27E-23	0.315759321	6.42E-19
Rgs3	1.03E-26	0.315532231	1.56E-22
Txk	1.89E-21	0.315250721	2.84E-17

Gm8369	4.79E-32	0.313769668	7.20E-28
Gimap9	7.85E-20	0.313729222	1.18E-15
Stat4	2.95E-19	0.308661479	4.44E-15
Gimap1os	1.61E-20	0.308619223	2.42E-16
Wipf1	3.24E-21	0.308538191	4.87E-17
Psmb9	3.14E-19	0.30650976	4.72E-15
Ccnd3	5.52E-18	0.306110708	8.29E-14
Il12rb2	2.64E-39	0.303741713	3.97E-35
St8sia4	2.82E-16	0.301645361	4.24E-12
Izumo1r	4.21E-31	0.301499256	6.34E-27
Cd2	5.57E-21	0.299527313	8.38E-17
Gm15472	1.30E-17	0.298367127	1.96E-13
Pde2a	1.64E-32	0.297013134	2.47E-28
Adgre5	1.39E-20	0.295683083	2.10E-16
Coro1a	5.98E-30	0.295315433	9.00E-26
P2rx7	1.48E-20	0.294585775	2.23E-16
Tnfrsf18	1.38E-19	0.292494223	2.08E-15
Isg15	3.20E-16	0.290981013	4.81E-12
Ccr5	1.61E-29	0.28798405	2.42E-25
Abcb1a	2.36E-30	0.286948318	3.55E-26
Entpd1	3.05E-30	0.286122415	4.59E-26
Il10rb	7.43E-24	0.285783291	1.12E-19
Fyn	9.51E-15	0.284654942	1.43E-10
Gm42418	2.02E-05	0.284213865	0.30331792
Foxn3	7.78E-14	0.283414944	1.17E-09
Abcb9	6.24E-25	0.282937249	9.38E-21
Sytl2	6.93E-26	0.282198851	1.04E-21
Agfg1	2.40E-18	0.281263035	3.62E-14
Tra2b	4.72E-17	0.278012371	7.10E-13
Ikzf1	4.59E-17	0.276026953	6.91E-13
Trip4	1.86E-16	0.27543319	2.80E-12
Tbx21	3.42E-22	0.272881642	5.14E-18
Ifi47	2.29E-17	0.272183528	3.45E-13
Cd27	1.86E-18	0.271515842	2.80E-14
H2-Q6	5.22E-18	0.271411969	7.85E-14
Lef1	1.06E-18	0.271026675	1.60E-14
Gimap8	1.08E-14	0.27095368	1.62E-10
Ccr9	1.94E-17	0.26978182	2.91E-13
Tmsb10	1.12E-42	0.267255311	1.68E-38
Txnip	6.51E-11	0.265278663	9.79E-07
Ifi208	9.43E-15	0.255955262	1.42E-10
Tm6sf1	1.12E-16	0.255427394	1.69E-12
Plaat3	1.93E-17	0.253240685	2.90E-13
Gimap1	1.96E-15	0.252725622	2.95E-11
Myh9	3.53E-13	-0.2530775	5.32E-09
Ppm1k	6.53E-20	-0.25437914	9.83E-16
B3galt2	7.54E-24	-0.25611804	1.13E-19
Pfkp	1.91E-13	-0.25811498	2.87E-09
Top2b	9.72E-12	-0.26029373	1.46E-07
Jund	8.18E-13	-0.26083369	1.23E-08
Oaz1	7.02E-31	-0.26104718	1.06E-26
Lpcat3	8.01E-23	-0.26133181	1.20E-18
Dennd4c	1.72E-17	-0.26134142	2.59E-13
Anxa5	1.88E-23	-0.26232159	2.82E-19
Nfkb1	1.73E-18	-0.26254398	2.61E-14
Epas1	1.14E-29	-0.26382556	1.71E-25
Lsp1	1.56E-20	-0.26461788	2.35E-16
Ier2	5.06E-12	-0.26707996	7.61E-08
Kdm6b	1.94E-18	-0.26773548	2.91E-14
Pkp3	1.17E-15	-0.26805624	1.76E-11
Cysltr1	5.28E-33	-0.26836101	7.93E-29
Plec	2.82E-14	-0.26850905	4.24E-10
Scp2	1.17E-19	-0.26884252	1.76E-15
Itm2b	9.42E-26	-0.26916518	1.42E-21
Nfatc3	1.30E-16	-0.2697053	1.96E-12
Snx2	2.00E-19	-0.27277426	3.01E-15
Acsbg1	3.91E-17	-0.27478859	5.89E-13
Ppp1r14b	7.65E-18	-0.27519261	1.15E-13
Crtc3	1.60E-20	-0.27555735	2.41E-16
Slc25a24	1.54E-26	-0.2761185	2.32E-22
Acat1	1.16E-21	-0.28013048	1.74E-17
Arf5	2.22E-22	-0.28165641	3.34E-18
Ahr	4.87E-28	-0.28183479	7.32E-24
Nt5e	3.00E-21	-0.28237767	4.51E-17
Cst3	2.33E-23	-0.28273148	3.50E-19
Kcnk1	7.37E-32	-0.2832279	1.11E-27
Mdfic	4.65E-20	-0.28379312	6.99E-16
Cd44	2.13E-16	-0.28443799	3.20E-12
Man2a2	1.41E-22	-0.28495415	2.11E-18
Elk3	9.66E-24	-0.28511033	1.45E-19
Nebl	7.13E-36	-0.28513788	1.07E-31
Gm17745	1.10E-31	-0.28777972	1.66E-27
Gabbr1	6.28E-27	-0.28908882	9.45E-23

Uhrf1bp1l	2.54E-22	-0.28926193	3.81E-18
Ssbp3	7.24E-22	-0.28930198	1.09E-17
Plcb4	1.76E-34	-0.28945607	2.64E-30
Eif4e3	8.50E-25	-0.28993314	1.28E-20
Znrf1	7.05E-21	-0.29059826	1.06E-16
Ikzf3	4.45E-18	-0.29099182	6.70E-14
Dap	1.83E-21	-0.29182013	2.75E-17
Atp1a1	1.12E-22	-0.29201252	1.69E-18
Rilpl2	1.16E-17	-0.29408186	1.74E-13
Atf6	2.64E-28	-0.2944613	3.98E-24
Nr4a1	3.11E-22	-0.29448454	4.67E-18
Xlr4a	9.47E-26	-0.29458923	1.43E-21
Neat1	5.22E-14	-0.29558188	7.86E-10
Chd4	1.64E-19	-0.29694753	2.46E-15
Clcn3	4.22E-22	-0.29710445	6.34E-18
Dusp1	3.55E-15	-0.29754122	5.34E-11
St3gal6	2.60E-18	-0.29831228	3.90E-14
B930036N	7.44E-19	-0.30055807	1.12E-14
Prkx	1.05E-22	-0.30113287	1.57E-18
Ppp1r14c	2.55E-39	-0.30191254	3.84E-35
Sptssa	4.15E-20	-0.30258094	6.24E-16
Il17a	2.31E-19	-0.30318626	3.48E-15
Avpi1	1.44E-40	-0.30697584	2.16E-36
Galnt1	1.15E-22	-0.31195539	1.73E-18
Cox7c	4.44E-35	-0.31317919	6.68E-31
Crip1	2.61E-06	-0.31472359	0.039324793
Selenot	4.28E-24	-0.31633495	6.43E-20
5830411N(	1.36E-22	-0.31774325	2.05E-18
Myo1e	1.26E-36	-0.31963228	1.90E-32
Rnf19b	1.40E-26	-0.32230616	2.11E-22
Camk2n1	1.36E-31	-0.32437441	2.04E-27
S1pr1	6.43E-33	-0.32479779	9.67E-29
Emp1	6.45E-34	-0.32734268	9.71E-30
Prdx6	8.69E-23	-0.32747092	1.31E-18
Odc1	2.03E-16	-0.32773404	3.05E-12
Plxnd1	1.29E-46	-0.32830092	1.94E-42
Slc38a1	2.32E-20	-0.3283548	3.49E-16
Tnfrsf25	1.93E-32	-0.32954757	2.90E-28
Smco4	1.78E-35	-0.33387329	2.68E-31
Fam83a	1.23E-51	-0.33453412	1.85E-47
H2afz	1.25E-25	-0.33554134	1.88E-21
Prelid2	3.19E-48	-0.33664877	4.80E-44
Gm20186	1.10E-15	-0.34119842	1.65E-11
AC160336.	2.53E-09	-0.34529025	3.80E-05
Kif1b	1.94E-31	-0.34688001	2.92E-27
Psap	1.62E-30	-0.34812844	2.44E-26
Rgs10	5.57E-29	-0.35024632	8.38E-25
Zbtb16	4.29E-26	-0.35078618	6.46E-22
Itgae	7.50E-45	-0.35087377	1.13E-40
Sik1	2.99E-32	-0.35197642	4.50E-28
Prr13	1.53E-28	-0.35373647	2.29E-24
Parp1	2.22E-33	-0.35625477	3.34E-29
Flna	1.51E-30	-0.36279412	2.28E-26
Tcrg-C1	1.62E-27	-0.36334221	2.44E-23
Atf3	5.24E-23	-0.3643814	7.87E-19
Cyb5a	1.87E-33	-0.36441221	2.81E-29
Mtss1	1.79E-58	-0.36489372	2.69E-54
Plin3	4.71E-45	-0.36507705	7.08E-41
Fam129a	1.49E-32	-0.36776897	2.24E-28
Fryl	9.05E-28	-0.36950315	1.36E-23
Il2ra	8.51E-27	-0.37142598	1.28E-22
Smox	1.96E-48	-0.37241855	2.95E-44
Cd69	4.19E-11	-0.3735024	6.31E-07
Slc25a4	9.22E-36	-0.37693584	1.39E-31
Laptm5	2.86E-51	-0.38626798	4.30E-47
Timp2	5.99E-42	-0.38705461	9.01E-38
Gadd45b	1.87E-22	-0.38707703	2.82E-18
Atp1b3	2.25E-30	-0.38825656	3.38E-26
Klf2	3.40E-14	-0.38826326	5.12E-10
Tspo	1.91E-43	-0.38921223	2.88E-39
Comt	2.24E-36	-0.3935918	3.36E-32
Egr1	4.40E-08	-0.395337	0.000661902
Aqp3	2.27E-54	-0.39675386	3.42E-50
Slco3a1	7.16E-40	-0.40125963	1.08E-35
Hspa5	5.24E-15	-0.40233522	7.88E-11
Ncor1	4.33E-39	-0.40359018	6.51E-35
Ramp3	2.10E-55	-0.40361154	3.16E-51
Lmna	1.11E-37	-0.40406931	1.67E-33
Stk24	6.97E-35	-0.40449719	1.05E-30
F2r	3.94E-25	-0.40683056	5.93E-21
Ly6g5b	2.37E-39	-0.41047263	3.56E-35
Wls	3.70E-44	-0.41428154	5.56E-40
Coro2b	4.17E-66	-0.41578352	6.27E-62

Atp2b1	8.79E-26	-0.4161534	1.32E-21
Smpdl3a	1.70E-32	-0.41655435	2.56E-28
Smap2	2.12E-40	-0.42270551	3.20E-36
Rara	2.04E-50	-0.4244137	3.07E-46
Mgat5	1.51E-47	-0.42786852	2.28E-43
Itgb7	1.18E-38	-0.43023323	1.77E-34
Ero1l	9.03E-49	-0.4354428	1.36E-44
Clint1	4.01E-38	-0.43567615	6.03E-34
Pim1	1.43E-25	-0.43804049	2.15E-21
Ccr6	2.49E-53	-0.43895091	3.75E-49
Ccr2	3.37E-34	-0.44304126	5.07E-30
Gm2a	6.56E-53	-0.44391202	9.86E-49
Nrip1	7.72E-28	-0.44391202	1.16E-23
Tbl1x	9.57E-43	-0.44454955	1.44E-38
Tax1bp1	7.99E-44	-0.44800274	1.20E-39
Rarg	4.47E-52	-0.46113076	6.73E-48
Cxcl10	1.77E-15	-0.46246978	2.67E-11
Emp3	7.22E-42	-0.46544133	1.09E-37
Il17re	4.28E-71	-0.46943484	6.44E-67
Pi4k2a	5.76E-55	-0.4735098	8.67E-51
Sdc1	1.04E-71	-0.47440394	1.56E-67
Rora	1.63E-38	-0.47721389	2.44E-34
1810058I2	1.02E-43	-0.47844669	1.53E-39
Nr1d1	2.00E-63	-0.48248692	3.01E-59
Tmem64	3.03E-36	-0.48379064	4.56E-32
Rnf144a	4.44E-58	-0.4850746	6.67E-54
Il23r	3.59E-80	-0.48720321	5.40E-76
Mmp25	3.93E-72	-0.49536051	5.91E-68
Furin	1.05E-37	-0.51001035	1.58E-33
Ifngr1	1.02E-22	-0.51212677	1.54E-18
Nme2	4.57E-56	-0.52329589	6.87E-52
Ahnak	1.55E-39	-0.5349399	2.33E-35
S100a10	1.08E-69	-0.54872532	1.62E-65
Sept11	1.79E-46	-0.55983487	2.70E-42
Tagln2	1.11E-51	-0.56560067	1.67E-47
Kcnc1	2.68E-82	-0.56727866	4.03E-78
Blk	1.28E-70	-0.56758753	1.93E-66
Il1r1	1.02E-88	-0.58245275	1.54E-84
Jaml	1.25E-57	-0.59004551	1.89E-53
Rexo2	7.04E-61	-0.59009475	1.06E-56
Ikzf2	9.96E-68	-0.61413856	1.50E-63
Ltb4r1	4.63E-89	-0.62118619	6.96E-85
Tcf7	7.32E-61	-0.62892547	1.10E-56
Actn2	6.10E-82	-0.63305389	9.17E-78
Klf4	7.16E-44	-0.639127	1.08E-39
Nfkbia	1.22E-38	-0.64023757	1.84E-34
Hk2	2.46E-64	-0.64911441	3.70E-60
Gpx1	2.18E-65	-0.66794979	3.28E-61
Gclc	1.41E-82	-0.6765166	2.12E-78
Gpr183	1.36E-76	-0.68957782	2.04E-72
Rgcc	2.00E-77	-0.69245557	3.01E-73
Rorc	1.49E-112	-0.70547702	2.25E-108
Lgals3	5.16E-56	-0.75709959	7.76E-52
S100a11	1.62E-105	-0.75779033	2.44E-101
Il18r1	5.01E-86	-0.79661781	7.53E-82
Vim	3.04E-68	-0.80672474	4.57E-64
Maf	2.31E-83	-0.82377644	3.47E-79
Icos	1.29E-93	-0.84929906	1.94E-89
Lgals1	1.41E-74	-0.87083883	2.12E-70
Emb	5.46E-100	-0.88094446	8.22E-96
Serpinb1a	1.71E-121	-0.88710767	2.57E-117
Zfp36l1	3.73E-90	-0.91956925	5.60E-86
Selenop	1.79E-103	-0.93176603	2.69E-99
Zfp36	5.04E-72	-0.9326285	7.58E-68
Jun	1.33E-64	-0.97925466	2.00E-60
Igf1r	9.07E-145	-0.98437553	1.36E-140
Capg	2.80E-134	-0.99187641	4.21E-130
Pxdc1	1.31E-145	-1.00853169	1.98E-141
Fosb	3.63E-58	-1.07188237	5.47E-54
Ramp1	2.63E-143	-1.13488387	3.95E-139
Lmo4	8.83E-118	-1.15982006	1.33E-113
Fos	1.63E-70	-1.17535396	2.45E-66
Tmem176b	2.69E-145	-1.19317604	4.05E-141
Il7r	5.20E-159	-1.30997561	7.82E-155
Tmem176a	1.96E-158	-1.31816875	2.95E-154
S100a6	8.09E-110	-1.3983366	1.22E-105
S100a4	5.04E-158	-1.90979055	7.58E-154

Supplementary Table 2

List of differentially expressed genes between MAIT cells from naïve mice (clusters 5 and 7) and MAIT at days 13 and 40 post infection (clusters 0, 1, 2, 3, 4, 6, 8, 9, 10, 11, 13), related to Figure 5D.
Statistical analysis was performed using two-sided wilcoxon rank sum test implemented in Seurat v3 FindMarkers() function.
Positive log fold-change: Upregulated in Clusters 5 and 7; Negative log fold-change: Downregulated in Clusters 5 and 7
(avg_logFC: average log-transformed fold-change, p.value: raw p-value, p_val_adj: p-value adjusted for multiple comparisons (bonferroni correction))

	p_val	avg_logFC	p_val_adj
Ccl5	0	1.331523632	0
Ccl4	7.45E-63	0.983368879	1.12E-58
Bhlhe40	1.61E-266	0.858535825	2.41E-262
Plac8	1.12E-250	0.856143868	1.68E-246
Ifng	6.67E-194	0.849745433	1.00E-189
Rgs1	8.08E-114	0.846912747	1.21E-109
Furin	8.01E-63	0.775504023	1.20E-58
Nkg7	0	0.749851565	0
Junb	4.40E-289	0.73562843	6.61E-285
Itga4	2.48E-180	0.694160002	3.73E-176
Crip1	4.23E-227	0.675667043	6.36E-223
Ly6a	5.44E-203	0.659121878	8.19E-199
Ly6c2	3.91E-120	0.637271616	5.88E-116
Btg2	8.45E-156	0.615091934	1.27E-151
Itga1	7.08E-165	0.605362928	1.07E-160
Gm36723	5.90E-144	0.590973683	8.87E-140
H2-Q7	1.53E-240	0.545770474	2.30E-236
AW11201C	6.06E-187	0.530989544	9.11E-183
Ctla2a	8.48E-117	0.523947992	1.28E-112
Nabp1	9.13E-136	0.516995483	1.37E-131
Ms4a4b	1.26E-161	0.514195619	1.90E-157
Gimap7	1.45E-173	0.513361201	2.19E-169
Itgb1	4.35E-106	0.49637688	6.54E-102
Anxa2	3.75E-150	0.488768349	5.64E-146
Klf6	3.31E-53	0.487504961	4.97E-49
Ccr5	3.12E-126	0.479141087	4.70E-122
Gm14718	7.66E-79	0.47623338	1.15E-74
Tnfaip3	5.23E-108	0.475091169	7.87E-104
Cd52	1.63E-246	0.448916792	2.46E-242
Serpinb9	2.36E-89	0.448484196	3.56E-85
Zeb2	4.53E-98	0.444570696	6.81E-94
Jund	1.39E-95	0.432638456	2.09E-91
Ctsd	3.53E-133	0.429685394	5.31E-129
Ctsw	2.45E-132	0.427164169	3.68E-128
Dusp5	7.81E-77	0.423635828	1.18E-72
Gm26532	9.90E-52	0.422184267	1.49E-47
Hcst	1.44E-150	0.413948281	2.16E-146
Epsti1	1.50E-122	0.412853752	2.25E-118
Serpina3g	1.59E-78	0.408984239	2.40E-74
Gm8369	4.44E-91	0.407288094	6.68E-87
Itgal	1.63E-133	0.406680153	2.45E-129
Gimap4	1.65E-118	0.391813684	2.48E-114
Traf1	3.31E-77	0.391791091	4.97E-73
Dnajc15	4.90E-105	0.388975986	7.38E-101
Sh3bgrl3	5.48E-164	0.386774641	8.25E-160
AA467197	8.18E-108	0.3790105	1.23E-103
Gem	2.73E-55	0.377067656	4.11E-51
Rbpj	9.85E-69	0.376526756	1.48E-64
Klrb1f	3.42E-77	0.373488275	5.15E-73
Rbm3	5.29E-105	0.368773101	7.96E-101
Chsy1	1.02E-88	0.363147823	1.54E-84
H2-K1	9.17E-185	0.363013195	1.38E-180
Zfp36l2	7.86E-38	0.356028093	1.18E-33
Spn	1.70E-75	0.355666485	2.55E-71
Ms4a6b	6.02E-96	0.35470147	9.05E-92
Btg1	2.73E-117	0.353421235	4.11E-113
Klrg1	7.35E-75	0.342393777	1.11E-70
H2-D1	8.23E-188	0.341892288	1.24E-183
Gm15472	5.88E-78	0.337176878	8.85E-74
Itm2b	3.71E-142	0.337010608	5.58E-138
Efhd2	1.28E-78	0.334436604	1.92E-74
Txn1	1.62E-88	0.333448604	2.43E-84
Cd7	1.86E-42	0.332104158	2.79E-38
Pnrc1	2.74E-95	0.329969255	4.12E-91
Arap2	3.85E-65	0.326174072	5.78E-61
Pfn1	6.45E-149	0.325906013	9.70E-145
Nr4a2	1.04E-49	0.325614777	1.57E-45
Gimap6	3.29E-89	0.320086573	4.95E-85
H3f3b	1.07E-73	0.316531055	1.61E-69
Gramd3	1.72E-70	0.316528945	2.58E-66
Ccr2	1.37E-63	0.314050209	2.06E-59
Gmfg	2.05E-84	0.313288613	3.09E-80
Il18rap	4.60E-55	0.311540072	6.92E-51
Lat	2.75E-75	0.309900857	4.14E-71
Aopep	8.35E-74	0.309879493	1.26E-69
Cd160	9.71E-50	0.308163909	1.46E-45
Slc38a2	3.75E-45	0.307879209	5.64E-41
Ptprc	1.55E-105	0.303037382	2.33E-101
Rac2	3.32E-119	0.301121551	5.00E-115
Gng2	4.51E-64	0.290679137	6.79E-60
Rora	1.75E-53	0.288684905	2.62E-49
Hif1a	1.29E-50	0.287913592	1.94E-46
Slamf1	4.63E-61	0.287775249	6.96E-57
Fgl2	3.67E-53	0.287178653	5.52E-49
Gapdh	2.76E-79	0.286679085	4.15E-75
Trat1	1.87E-55	0.286204775	2.81E-51
Themis	8.61E-52	0.281742699	1.29E-47
Sp100	5.62E-63	0.281047117	8.46E-59
BE692007	2.87E-54	0.27826908	4.31E-50
Ahnak	1.68E-68	0.276306725	2.52E-64
Hopx	4.41E-61	0.274728334	6.64E-57
Gm19585	3.08E-57	0.273919	4.64E-53
Fasl	1.21E-52	0.273597019	1.83E-48
Slamf7	5.69E-56	0.270081167	8.56E-52
Actb	4.17E-99	0.264728124	6.27E-95
Fxyd5	3.08E-76	0.260308036	4.63E-72
Casp1	1.27E-74	0.259240626	1.91E-70
Cd40lg	1.31E-39	0.258175372	1.97E-35

Cyba	9.97E-73	0.257234045	1.50E-68
Slc20a1	1.01E-42	0.257061396	1.52E-38
Cd6	5.04E-39	0.254007857	7.59E-35
B2m	1.00E-119	0.252649261	1.50E-115
Cd48	1.33E-44	0.251567667	1.99E-40
Bcl2	8.37E-15	-0.25114997	1.26E-10
Kcnc1	2.94E-112	-0.25128651	4.43E-108
Smco4	2.70E-88	-0.2522543	4.06E-84
Stip1	2.47E-58	-0.2523761	3.71E-54
Filip1l	7.98E-60	-0.25246758	1.20E-55
Il1r1	1.96E-100	-0.25268201	2.95E-96
Rpl15	3.59E-97	-0.25291864	5.40E-93
Il2rb	8.56E-11	-0.25684868	1.29E-06
Rpl23	3.13E-168	-0.25709092	4.71E-164
Sesn1	3.95E-86	-0.2588047	5.94E-82
Klf4	2.11E-42	-0.259657	3.17E-38
Sep-11	2.38E-31	-0.26049948	3.57E-27
Rpl35a	2.01E-153	-0.26098577	3.02E-149
Rgcc	8.69E-39	-0.2641554	1.31E-34
Satb1	1.67E-33	-0.26668528	2.51E-29
Rpl32	1.34E-142	-0.26841031	2.01E-138
Rpl31	9.85E-69	-0.26934723	1.48E-64
Rps12	8.15E-139	-0.27270748	1.23E-134
Rps29	1.42E-195	-0.27303473	2.14E-191
mt-Nd4	2.66E-106	-0.27406006	4.00E-102
Hspd1	1.17E-50	-0.27625815	1.76E-46
Ppp1r14b	1.61E-49	-0.27740987	2.41E-45
Rps27a	7.20E-206	-0.28188914	1.08E-201
Rplp1	1.85E-194	-0.28189346	2.78E-190
Rps8	2.79E-167	-0.28862878	4.19E-163
Tespa1	4.90E-76	-0.29097316	7.38E-72
Hmgn1	5.18E-81	-0.29119683	7.78E-77
Blk	8.77E-281	-0.29425287	1.32E-276
Klre1	3.15E-20	-0.29450032	4.74E-16
Rpl13	6.34E-206	-0.2970553	9.53E-202
Rps18	6.59E-202	-0.30184684	9.91E-198
Hspe1	1.30E-68	-0.30670422	1.96E-64
Gclc	5.32E-64	-0.30699899	8.01E-60
Rps19	4.36E-155	-0.31015291	6.55E-151
Rps17	3.19E-105	-0.31116315	4.80E-101
Bzw2	6.81E-77	-0.3122314	1.02E-72
Wls	7.80E-188	-0.31264579	1.17E-183
Nt5e	1.33E-194	-0.31278522	2.01E-190
Rgs10	5.48E-108	-0.31704789	8.23E-104
Capg	5.16E-48	-0.3188421	7.77E-44
Zfp36	2.09E-20	-0.31903711	3.14E-16
Klf3	4.58E-99	-0.32292989	6.89E-95
Socs2	3.09E-109	-0.3251295	4.64E-105
Rorc	2.84E-146	-0.33028151	4.28E-142
mt-Nd5	1.28E-88	-0.3315706	1.93E-84
Ikzf2	1.93E-81	-0.33469453	2.90E-77
Ndufa4	2.09E-103	-0.33928827	3.15E-99
Rpl28	8.38E-133	-0.3419528	1.26E-128
Sh3bp5	0	-0.34405966	0
Rps26	2.23E-149	-0.34800667	3.35E-145
Rexo2	2.91E-64	-0.34976649	4.37E-60
Eef1b2	7.97E-153	-0.35066637	1.20E-148
Zfp36l1	3.09E-33	-0.35096693	4.65E-29
Rnf144a	0	-0.35135744	0
Pde3b	3.40E-85	-0.36225356	5.11E-81
mt-Nd4l	2.11E-87	-0.3650245	3.18E-83
Ifngr1	8.53E-45	-0.36766878	1.28E-40
Rpl36a	8.50E-178	-0.37190458	1.28E-173
Gpx1	1.29E-65	-0.37677364	1.94E-61
Klrd1	4.99E-26	-0.37709383	7.50E-22
Rps20	4.50E-203	-0.38082747	6.77E-199
Maf	1.17E-48	-0.38101466	1.76E-44
Igf1r	3.73E-79	-0.38311536	5.61E-75
Dnaja1	6.14E-115	-0.40506797	9.23E-111
Klrk1	2.87E-59	-0.41245483	4.31E-55
Fos	1.59E-31	-0.4464615	2.39E-27
Lyst	8.77E-56	-0.45205568	1.32E-51
Tmem64	2.41E-122	-0.4679383	3.62E-118
Selenop	4.07E-89	-0.47246217	6.12E-85
Fosb	3.76E-24	-0.49141335	5.66E-20
Rpl35	8.82E-277	-0.56213877	1.33E-272
Lars2	5.35E-223	-0.57304114	8.05E-219
Pxdc1	1.22E-251	-0.5857083	1.83E-247
Ramp1	2.03E-132	-0.63373636	3.06E-128
Ifi27l2a	6.87E-116	-0.64005328	1.03E-111
mt-Atp8	1.37E-258	-0.66637257	2.06E-254
Tmem176b	4.57E-244	-0.77029169	6.88E-240
Gm42418	0	-0.80257617	0
AY036118	0	-0.81709374	0
Tmem176a	9.43E-308	-0.89586308	1.42E-303
Lmo4	6.45E-248	-0.93930661	9.70E-244
Il7r	1.12E-171	-0.95946759	1.69E-167

Supplementary Table 3. Survival of cytokine-deficient mice with *F. tularensis* LVS infection.

**Mouse strain**	***F. tularensis* LVS infection dose (CFU, i.v.)**						
	**10**	**100**	**400**	**1600**	**5000**	**10000**	**20000**
WT (C57BL/6)						20*/20¶	20/20
Tnf$^{-/-}$	0/10	0/10	0/8	0/5			
Ifnγ$^{-/-}$	0/10	0/10	0/5	0/5			
Gm-csf$^{-/-}$		5/5	10/10	6/10	0/10	0/10	
Il-17$^{-/-}$					10/10	0/10	0/10

Mice proficient or deficient in cytokines known to be produced by MAIT cells were infected with indicated doses of *F. tularensis* LVS (i.v.) and monitored for survival (human endpoints as described in methods). *= number of mice survived, ¶= number of mice infected. Empty cells = not done.
