# Supplementary material for: Francisella tularensis induces Th1 like MAIT cells conferring protection against systemic and local infection
Source: Nat Commun. 2021 Jul 16;12:4355. doi: 10.1038/s41467-021-24570-2 (PMC8285429; doi:10.1038/s41467-021-24570-2)
Supplement: Supplementary file 2 — Reporting summary [file 41467_2021_24570_MOESM2_ESM.pdf]

nature research

Corresponding author(s): Alexandra Corbett, Zhenjun Chen, James McCluskey

Last updated by author(s): 2021/5/13

Reporting Summary

Nature Research wishes to improve the reproducibility of the work that we publish. This form provides structure for consistency and transparency in reporting. For further information on Nature Research policies, see our Editorial Policies and the Editorial Policy Checklist.

Statistics

For all statistical analyses, confirm that the following items are present in the figure legend, table legend, main text, or Methods section.

n/a	Confirmed	
☐	☒	The exact sample size (n) for each experimental group/condition, given as a discrete number and unit of measurement
☐	☒	A statement on whether measurements were taken from distinct samples or whether the same sample was measured repeatedly
☐	☒	The statistical test(s) used AND whether they are one- or two-sided<br>*Only common tests should be described solely by name; describe more complex techniques in the Methods section.*
☐	☒	A description of all covariates tested
☐	☒	A description of any assumptions or corrections, such as tests of normality and adjustment for multiple comparisons
☐	☒	A full description of the statistical parameters including central tendency (e.g. means) or other basic estimates (e.g. regression coefficient) AND variation (e.g. standard deviation) or associated estimates of uncertainty (e.g. confidence intervals)
☐	☒	For null hypothesis testing, the test statistic (e.g. F, t, r) with confidence intervals, effect sizes, degrees of freedom and P value noted<br>*Give P values as exact values whenever suitable.*
☒	☐	For Bayesian analysis, information on the choice of priors and Markov chain Monte Carlo settings
☒	☐	For hierarchical and complex designs, identification of the appropriate level for tests and full reporting of outcomes
☒	☐	Estimates of effect sizes (e.g. Cohen's d, Pearson's r), indicating how they were calculated

Our web collection on statistics for biologists contains articles on many of the points above.

Software and code

Policy information about availability of computer code

Data collection	Cytometry data were collected with Diva version 7 BD Biosciences.
Data analysis	FlowJo V10 was used for flow cytometric data analysis, scatter plot3D, Statistical tests were performed using Prism GraphPad (version 9.1). 3D projections of UMAP were created by the scatter plot3D version 0.3-41 R package. Analysis of single-cell RNA-seq data was performed with 10x Genomics Cell Ranger (version 3.1.0) and Seurat (version 3.1.5).

For manuscripts utilizing custom algorithms or software that are central to the research but not yet described in published literature, software must be made available to editors and reviewers. We strongly encourage code deposition in a community repository (e.g. GitHub). See the Nature Research guidelines for submitting code & software for further information.

Data

Policy information about availability of data

All manuscripts must include a data availability statement. This statement should provide the following information, where applicable:

- Accession codes, unique identifiers, or web links for publicly available datasets
- A list of figures that have associated raw data
- A description of any restrictions on data availability

All relevant data from this study, including raw flow-cytometry data, are available from the corresponding authors upon reasonable request. The full raw single-cell RNA sequencing data generated has been submitted to ArrayExpress database (http://www.ebi.ac.uk/arrayexpress) ArrayExpress (lOx Genomics scRNA-seq data: accession number E-MTAB-10239.

Field-specific reporting

Please select the one below that is the best fit for your research. If you are not sure, read the appropriate sections before making your selection.

[x] Life sciences [ ] Behavioural & social sciences [ ] Ecological, evolutionary & environmental sciences

For a reference copy of the document with all sections, see nature.com/documents/nr-reporting-summary-flat.pdf

Life sciences study design

All studies must disclose on these points even when the disclosure is negative.

Sample size	For functional assays. All experiments were performed at least twice (independent experiments) using duplicate or triplicate samples, with similar results obtained from independent experiments. Sample sizes were estimated based on previous extensive experience in the laboratory within similar studies.
Data exclusions	for RNAseq data exclusions of clusters based on quality controls are described in the methods
Replication	All experiments, except for RNA sequencing were performed at least twice with similar results obtained in all replication attempts.
Randomization	Mice or samples were randomly allocated to groups. All groups being compared were gender and age matched, with housing, feeding and husbandry matched to minimise covariates.
Blinding	Blinding was not applicable to the study as there were no subjective measures (e.g. scoring) used for analysis. Blinding was not possible during cytometry experiments as MAIT cells are absent from MR1-/- mice, and thus the species phenotype could not be concealed from the operator.

Reporting for specific materials, systems and methods

We require information from authors about some types of materials, experimental systems and methods used in many studies. Here, indicate whether each material, system or method listed is relevant to your study. If you are not sure if a list item applies to your research, read the appropriate section before selecting a response.

Materials & experimental systems

n/a	Involved in the study	
[ ]	[x]	Antibodies
[ ]	[x]	Eukaryotic cell lines
[x]	[ ]	Palaeontology and archaeology
[ ]	[x]	Animals and other organisms
[x]	[ ]	Human research participants
[x]	[ ]	Clinical data
[x]	[ ]	Dual use research of concern

Methods

n/a	Involved in the study	
[x]	[ ]	ChIP-seq
[ ]	[x]	Flow cytometry
[x]	[ ]	MRI-based neuroimaging

Antibodies

Antibodies used	Antibodies against murine CD45.2 (Clone 104, Cat #553772, FITC, 1:200), CD19 (clone 1D3, Cat #551001, PerCPcy5.5, 1:200), TCR (clone H57-597, Cat #553174, APC, 1:200 or Cat #553172, PE, 1:200), CD44 (clone IM7, Cat #612799, BUV737, 1:200 or Cat #740215, BUV395, 1:200), CD4 (clone GK1.5, Cat #552051, APC-Cy7, 1:200), IFN (clone XMG1.2, Cat #557649, PE-Cy7, 1:400), IL-17A (clone TC11-18H10, Cat #559502, PE, 1:200), GM-CSF (clone MP1-22E9, Cat #554406, PE, 1:200), TNF (clone MP6-XT22, Cat #557644, PE-Cy7, 1:200), Ly6G (clone 1A8, Cat #560601, PEcy7, 1:200), CD103 (clone M290, Cat #740238, BUV395, 1:200), CD11b (clone M1/70, Cat #557657, APC-Cy7, 1:200), Ly6C (clone AL-21, Cat #563011, BV605, 1:200), CD49a (clone Hα31/8, Cat #740375, BV605, 1:200), CD62L (clone MEL-14, Cat #565159, APC-R700, 1:400) and CD8β (clone:H35-17.2, Cat#747505, BV750, 1:400) were purchased from BD Biosciences (Franklin Lakes, NJ). Antibodies against CD8a (clone 53-6.7, #12-0081-83, PE, 1:1000), RORt (clone: B2D, #17-6981-82, APC, 1:200), T-bet (clone: 4B10, #25-5825-82, PE-Cy7, 1:200), CD49b (clone DX5, #25-5971-81, PE-Cy7, 1:200), IL-18Ra (clone P3TUNYA, #48-5183-82, eFluor 450, 1:400), CD69 (clone H1.2F3, #15-0691-82, PE-Cy5, 1:200) and CD45 (clone: 30-F11, #58-0451-82, AF532, 1:200) were purchased from eBioscience (San Diego, CA). Abs against F4/80 (clone BM8, Cat #123116, APC, 1:200), MHCII (clone M5/114, Cat #107631, BV421, 1:200), CD11c (clone N418, Cat #117308, PE, 1:200), NK1.1 (clone PK136, Cat #108731, BV421, 1:200) and CD127 (clone A7R34, Cat #135031, PE/Dazzle 594, 1:200), TCRβ (clone:H57-597, Cat#109246, APC/Fire 750, 1:400) and CD8α (clone:53-6.8, Cat#100782, Spark NIR685, 1:400) were purchased from BioLegend (San Diego, CA). Blocking Ab (26.5: anti human MR1 MoAb or 8F2.F9: anti-mouse MR1 MoAb) and isotype controls (3E12, 8A5), as well as MR1 tetramers, were prepared in house.
Validation	Validation<br>Antibody validation for the species - mice - is provided for all commercially available antibodies described above on the relevant manufacturer's website accessible via the catalogue numbers which are also provided above: bdbiosciences.com, biolegend.com and

thermofisher.com (for eBioscience).

Eukaryotic cell lines

Policy information about cell lines

Cell line source(s)	C1R-MR1 and Jurkat MAIT Cells lines have been previously described and are references in the manuscript. The parental Jurkat cell line was originally obtained from the ATCC: J.RT3-T3.5 (ATCC® TIB-153™). The parental C1R cell line has been described: Zemmour et al. J. Immunol 1992. 148(6):1941-1948
Authentication	No authentication of cell lines was conducted. The identity of cell lines (other than expression of transduced receptors, which was confirmed) is not considered relevant to the experimental comparisons conducted.
Mycoplasma contamination	Cell lines used have been tested and confirmed as mycoplasma negative prior to storage in Liquid N2
Commonly misidentified lines (See ICLAC register)	No commonly misidentified cell lines were used in the study.

Animals and other organisms

Policy information about studies involving animals; ARRIVE guidelines recommended for reporting animal research

Laboratory animals	Mus muscularis strains C57BL/6 and MR1-/- (on C57BL/6 background). Males and females aged 6-12 weeks. Mice were housed under standard SPF conditions in the Biological Research Facility of the Doherty Institute, using a 12-hour day/night light cycle with (light from 7am to 7pm and dark from 7pm to 7am), humidity of 40-70% and temperature of 19-22˚C.
Wild animals	the study did not involve wild animals
Field-collected samples	the study did not involve samples collected from the field
Ethics oversight	all experiments on mice were conducted following approval by the University of Melbourne Animal Ethics Committee

Note that full information on the approval of the study protocol must also be provided in the manuscript.

Flow Cytometry

Plots

Confirm that:

- [x] The axis labels state the marker and fluorochrome used (e.g. CD4-FITC).
- [x] The axis scales are clearly visible. Include numbers along axes only for bottom left plot of group (a 'group' is an analysis of identical markers).
- [x] All plots are contour plots with outliers or pseudocolor plots.
- [x] A numerical value for number of cells or percentage (with statistics) is provided.

Methodology

Sample preparation	Single cell suspensions were prepared from mouse spleen, lungs, liver, kidneys and blood using manual dissection and collagenase digestion as fully described in the methods. Staining with antibodies and tetramers for surface markers and intracellular staining are described in the methods.
Instrument	LSRII or LSR Fortessa or Canto II (BD Biosciences) or Aurora (Cytek) flow cytometers or BD Aria III flow sorter.
Software	Diva software V7 (BD Biosciences) was used for acquisition of sample data. Data was analysed using FlowJo version 10 (Treestar).
Cell population abundance	MAIT cells were FACS sorted for adoptive transfer and for RNA seq. Post sort purity was assessed by staining using the same gating strategy and was >90% in each case.
Gating strategy	The gating strategy is described in Supplemental Figure 1 and related to all figures, except for Fig S6 and Fig S10, where experiment-specific gating strategies are provided.

- [x] Tick this box to confirm that a figure exemplifying the gating strategy is provided in the Supplementary Information.
